# A predator-prey interaction between a marine *Pseudoalteromonas* sp. and Gram-positive bacteria

**DOI:** 10.1038/s41467-019-14133-x

**Published:** 2020-01-15

**Authors:** Bai-Lu Tang, Jie Yang, Xiu-Lan Chen, Peng Wang, Hui-Lin Zhao, Hai-Nan Su, Chun-Yang Li, Yang Yu, Shuai Zhong, Lei Wang, Ian Lidbury, Haitao Ding, Min Wang, Andrew McMinn, Xi-Ying Zhang, Yin Chen, Yu-Zhong Zhang

**Affiliations:** 1 State Key Laboratory of Microbial Technology, Marine Biotechnology Research Center, Shandong University, Qingdao, 266237 China; 2 College of Marine Life Sciences, Institute for Advanced Ocean Study, Ocean University of China, Qingdao, 266003 China; 3 Laboratory for Marine Biology and Biotechnology, Pilot National Laboratory for Marine Science and Technology (Qingdao), Qingdao, 266373 China; 4 School of Life Sciences, University of Warwick, Coventry, UK; 5 SOA Key Laboratory for Polar Science, Polar Research Institute of China, Shanghai, 200136 China; 6 Institute for Marine and Antarctic Studies, University of Tasmania, Hobart, TAS Australia

**Keywords:** Microbial ecology, Marine microbiology, Marine biology

## Abstract

Predator-prey interactions play important roles in the cycling of marine organic matter. Here we show that a Gram-negative bacterium isolated from marine sediments (*Pseudoalteromonas* sp. strain CF6-2) can kill Gram-positive bacteria of diverse peptidoglycan (PG) chemotypes by secreting the metalloprotease pseudoalterin. Secretion of the enzyme requires a Type II secretion system. Pseudoalterin binds to the glycan strands of Gram positive bacterial PG and degrades the PG peptide chains, leading to cell death. The released nutrients, including PG-derived D-amino acids, can then be utilized by strain CF6-2 for growth. Pseudoalterin synthesis is induced by PG degradation products such as glycine and glycine-rich oligopeptides. Genes encoding putative pseudoalterin-like proteins are found in many other marine bacteria. This study reveals a new microbial interaction in the ocean.

## Abstract (web summary)

Predator-prey interactions play important roles in the cycling of marine organic matter. Here the authors show that a Gram-negative bacterium isolated from marine sediments can kill and feed on Gram-positive bacteria by secreting a peptidoglycan-degrading enzyme.

## Introduction

Bacteria are ubiquitous in marine ecosystems and play a vital role in nutrient recycling in the microbial loop^1^. Predation on bacteria and the accompanying mortality are important mechanisms for bacterial population control and nutrient recycling. While viruses and protists are known agents responsible for bacterial mortality, predatory bacteria may also contribute substantially to bacterial death, although there are only a few known examples from the ocean^2,3^. Arguably, the best studied predators of bacteria are *Bdellovibrio* and *Bdellovibrio*-like organisms (BALOs) that prey on a wide range of Gram-negative bacteria^4^. Like Gram-negative bacteria, Gram-positive bacteria are also widespread in the ocean^5–7^, comprising up to 14% and 25% of total bacterial cell counts in seawater and sediments, respectively^8^. It is unclear, however, whether Gram-positive marine bacteria can also be preyed on by bacteria.

Predatory bacteria actively hunt and kill their prey and consume their macromolecules as nutrients^9,10^. The strategies used by predatory bacteria to kill their prey bacteria vary from case to case; however, they generally include: (i) epibiotic predation, in which predators consume the prey from the outside^9^; (ii) endobiotic predation or direct invasion, in which an individual predatory cell secretes hydrolytic enzymes that perforate and modify the prey cell wall, in order to penetrate either into the periplasmic space or into the cytoplasm^9^; or (iii) group attack, in which a quorum of predators produce hydrolytic enzymes/secondary metabolites to degrade the prey cells^9,11^. The cell wall is thus usually a key target of attack by hydrolytic enzymes for predatory bacteria to kill their prey. Peptidoglycan (PG) is an important structural component of the bacterial cell wall, accounting for up to 90% of the cell wall in Gram-positive bacteria and 10% in Gram-negative bacteria. Structurally, PG is a network formed by linear glycan strands interconnected by peptide stems that are typically composed of amino acid residues l-Ala, d-Glu, l-Lys/diaminopimelate, and d-Ala, and are linked directly or through a short peptide bridge^12^. A certain degree of variation has been found either in the peptide stem, in the glycan strands, or in the position or composition of the peptide bridge^12^. Three main classes of PG hydrolases have been described; glycosidases cleave the bonds in the glycan strands, amidases cleave the bond between the glycan strand and the peptide chain, and endopeptidases cleave the bonds within the peptide chains^13^.

Here, we show that *Pseudoalteromonas* sp. strain CF6-2, a Gram-negative bacterium from a deep-sea sediment, can kill a wide range of Gram-positive bacteria by secreting a large quantity of the M23 metalloprotease pseudoalterin^14,15^, and thus degrading the PG in their cell wall. The released nutrients can then be utilized by strain CF6-2 for growth. Bioinformatics analyses suggest that genes encoding pseudoalterin-like proteins are found in other marine bacteria.

## Results

### Strain CF6-2 kills Gram-positive bacteria via secreted products

**Fig. 1:**
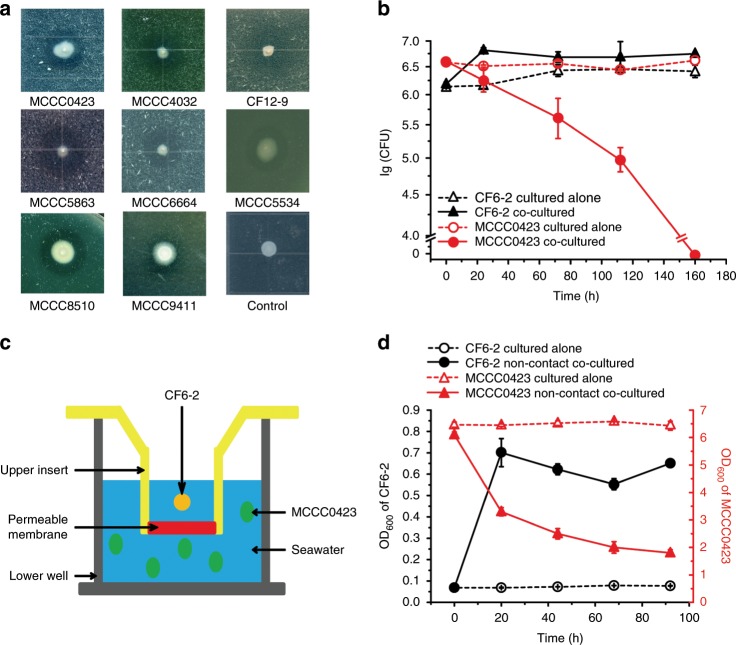
Predation of Gram-positive bacteria by strain CF6-2. **a** The killing effect of strain CF6-2 on eight Gram-positive marine bacteria on agar plates. Strain CF6-2 was spotted and grew on each plate containing the cells of the indicated strain at 20 °C for 3 days until a clear zone around CF6-2 colony formed. MCCC0423, *Staphylococcus warneri* MCCC1A00423 (peptide stem, Ae(q)Ka; peptide bridge, GGGGG)^12^; MCCC4032, *Micrococcus luteus* MCCC1A04032 (peptide stem, Ae(q)Ka/Ae(G)Ka; peptide bridge, EG/Ae(G)Ka)^16^; CF12-9, *Bacillus* sp. CF12-9 (peptide stem, Ae(q)Ka/AeKa/AeKa; peptide bridge, G/d(n)/Ad)^16^; MCCC5863, *Sporosarcina aquimarina* MCCC1A05863 (peptide stem, AeKa; peptide bridge, Ge)^16, 51^; MCCC6664, *Streptomyces* sp. MCCC1A06664 (peptide stem, Ae(q)pa; peptide bridge, G)^16, 52^. MCCC5534, *Salinibacterium amurskyense* MCCC1A05534 (PG-containing Lys, Orn, Ala, Gly, and Glu)^16, 53^; MCCC8510, *Exiguobacterium* sp. MCCC1A08510 (peptide stem, Ae(q)Ka; peptide bridge, G); MCCC9411, *Exiguobacterium profundum* MCCC 1A09411 (peptide stem, Ae(q)Ka; peptide bridge, G)^54^. Control, strain CF6-2 on an agar plate without any other bacteria. The PG chemotype of each strain is shown in brackets. The figure shows a representative of triplicate experiments. **b** The predation of strain CF6-2 on strain MCCC0423 when co-cultured in artificial seawater at 20 °C. **c** A schematic diagram depicting the experiment used to investigate the non-contact interaction between strains CF6-2 and MCCC0423. The Transwell^®^ permeable supports device used in this experiment is composed of an upper insert (colored in yellow and red) and a lower well (dark gray). The bottom of the upper insert is a permeable membrane (red) with a pore size of 0.4 μm that bacteria cannot pass through (Supplementary Fig. 2). **d** The killing effect of strain CF6-2 on strain MCCC0423 in the non-contact co-culture performed in the Transwell^®^ permeable supports device. The data in **b** and **d** are mean ± SD of triplicate experiments. Source data are provided as a Source Data file.

To analyze whether strain CF6-2 has antagonistic interactions with other marine bacteria, the interaction of strain CF6-2 with a variety of Gram-positive and Gram-negative marine bacteria on agar plates was investigated. Strain CF6-2 had no effect on the growth of several representative Gram-negative marine bacteria (Supplementary Fig. 1) but could inhibit the growth of eight Gram-positive marine bacterial strains of seven different chemotypes of PG, forming clear zones around its colonies on agar plates (Fig. 1a). We further tested the interaction of strain CF6-2 and a representative Gram-positive bacterium *Staphylococcus warneri* strain MCCC1A00423 (hereafter strain MCCC0423, from South China Sea sediments) in artificial seawater. Co-culturing of the two bacteria led to a strong reduction in the cell numbers of strain MCCC0423 over time and an increase in the cell numbers of strain CF6-2, indicating the ability of strain CF6-2 to prey on strain MCCC0423 cells for nutrients (Fig. 1b).

Because clear zones developed around the strain CF6-2 colonies in Fig. 1a, we hypothesized that strain CF6-2 inhibited the growth of Gram-positive bacteria, likely via its secretion products. To support this, an experiment was designed to examine the nature of non-contact interactions between strains CF6-2 and MCCC0423 in artificial seawater using a Transwell^®^ Permeable Supports device (Fig. 1c). In this device, organic molecules can pass through the permeable membrane (pore size, 0.4 μm) but the cells of strain CF6-2 or MCCC0423 cannot (Supplementary Fig. 2). In the non-contact co-culture experiment, the cell numbers of strain CF6-2 in the upper insert increased over time and the cell numbers of strain MCCC0423 in the lower well decreased over time (Fig. 1d). This result suggests firstly that some compound(s) secreted by strain CF6-2 passed through the permeable membrane into the lower well, leading to the death of strain MCCC0423, and secondly that the resultant nutrients of strain MCCC0423 are capable of supporting the growth of strain CF6-2.

### Pseudoalterin is involved in killing Gram-positive bacteria

**Fig. 2:**
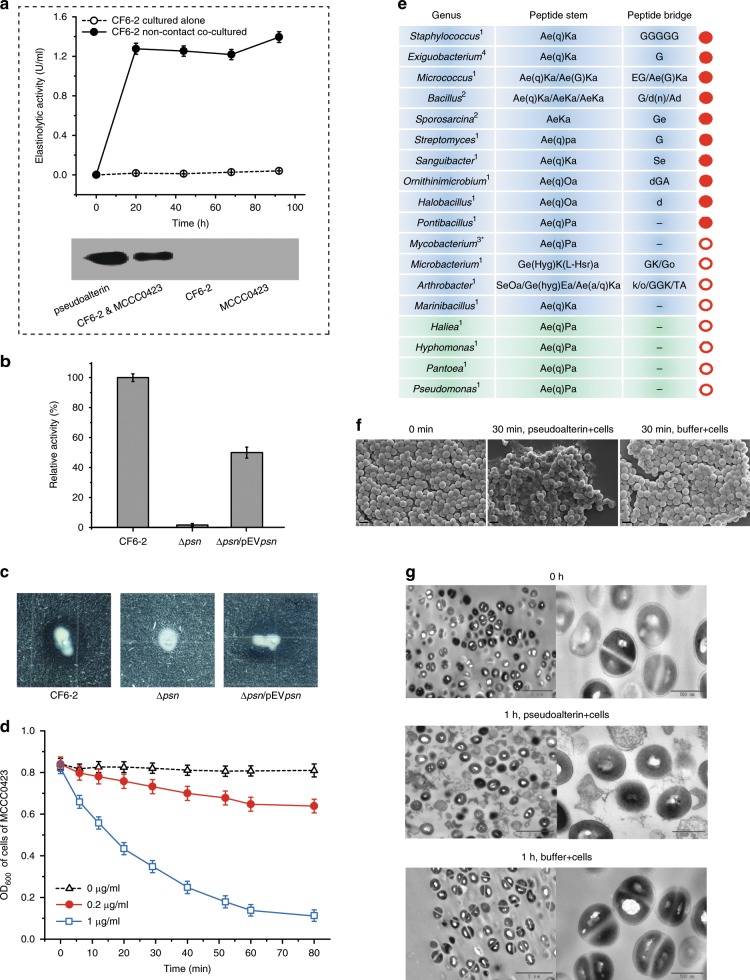
Gram-positive bacterial killing by pseudoalterin. **a** Detection of pseudoalterin secreted by strain CF6-2 in the non-contact co-culture. The upper panel shows the extracellular elastinolytic activity. The lower panel shows pseudoalterin production detected by western blot. Pseudoalterin has been shown to have significant elastinolytic activity because elastin is rich in Gly bonds^15^. **b** The extracellular elastinolytic activity of the Δ*psn* mutant and the complementary strain Δ*psn*/pEV*psn*. Values are normalized against the activity of the WT CF6-2 strain. **c** Abilities of the Δ*psn* mutant and the complementary strain Δ*psn*/pEV*psn* to prey strain MCCC0423. Strains CF6-2, Δ*psn*, and Δ*psn*/pEV*psn* were spotted and grew on plates containing the cells of strain MCCC0423 at 20 °C for 3 days until clear zones formed around the colonies of strains CF6-2 and Δ*psn*/pEV*psn*. **d** Time–kill microbial curves of pseudoalterin against cells of strain MCCC0423 at 25 °C. **e** Killing activity of pseudoalterin against marine bacteria with different PG chemotypes. The suspension of bacterial cells (OD_600_ = 0.8–1.0) was incubated at 25 °C for 120 min with 20 μg ml^−1^ of pseudoalterin in 20 mM Tris-HCI buffer (pH 9.0). Gram-positive bacteria are in blue background and Gram-negative bacteria in green background. The superscript figure after each genus indicates the number of tested strains in each genus. The PG chemotype of each genus is shown. The star indicates that the PG of these three strains is covered by a large amount of unusual lipids in the cell wall^21^. Solid red circles indicate pseudoalterin has killing activity and hollow red circles indicate pseudoalterin has no killing activity. The killing rate (%) of pseudoalterin against each strain is shown in Supplementary Table 1. **f** SEM observation of cells of strain MCCC0423 treated by pseudoalterin. Bars: 1 μm. Control, cells of strain MCCC0423 treated with buffer for 30 min. **g** TEM observation of cells of strain MCCC0423 treated by pseudoalterin. Bars: 2 μm in the upper pictures and 500 nm in the lower pictures. Source data are provided as a Source Data file.

The killing activity of the compound(s) secreted by strain CF6-2 in the non-contact co-culture of strains CF6-2 and MCCC0423 was abolished by heating (Supplementary Fig. 3a) or by an addition of 2 mM Zn^2+^ (Supplementary Fig. 3b). Since strain CF6-2 can secrete a large amount of the thermolabile protease pseudoalterin^14^ and because additional Zn^2+^ can inhibit the activity of pseudoalterin^15^, we therefore postulated that strain CF6-2 secreted pseudoalterin in the non-contact co-culture experiment. Indeed, this was supported by protease activity assays and western blot assays. Both the pseudoalterin protein and its activity were detected in the non-contact co-culture, none of which, however, was detectable in the culture of strain CF6-2 or strain MCCC0423 when cultured alone (Fig. 2a), suggesting that pseudoalterin production of strain CF6-2 was induced in the co-culture. In order to demonstrate the involvement of pseudoalterin in killing Gram-positive bacteria, we constructed a mutant of strain CF6-2, Δ*psn*, in which the pseudoalterin gene was knocked out. As expected, this mutant did not secrete pseudoalterin any more (Fig. 2b) and lost the ability to inhibit the growth of strain MCCC0423 cells (Fig. 2c), indicating the involvement of pseudoalterin in killing MCCC0423 cells. Moreover, in vitro experiments showed that recombinant pseudoalterin could kill the cells of strain MCCC0423, leading to a strong dose-dependent reduction of cell numbers over time (Fig. 2d). Recombinant pseudoalterin is also capable of killing 14 other Gram-positive marine bacteria representing 10 different chemotypes of PG (Fig. 2e).

### Pseudoalterin is active against Gram-positive bacterial PG

Next, we set out to uncover the mechanisms by which pseudoalterin kills the Gram-positive bacteria. Scanning electron microscopy (SEM) images shown in Fig. 2f demonstrated significant alteration of cell surface structure of strain MCCC0423 after pseudoalterin treatment. The dynamics of this interaction was observed using time-lapse atomic force microscope (AFM) and the movie presented in the Supplementary Video file (Supplementary Movie 1) showed gradual alteration and subsequent collapse of cell structure of strain MCCC0423 during pseudoalterin treatment. Indeed, we observed debris of individual cells of strain MCCC0423 (Fig. 2g) using high-resolution transmission electron microscopy (TEM).

**Fig. 3:**
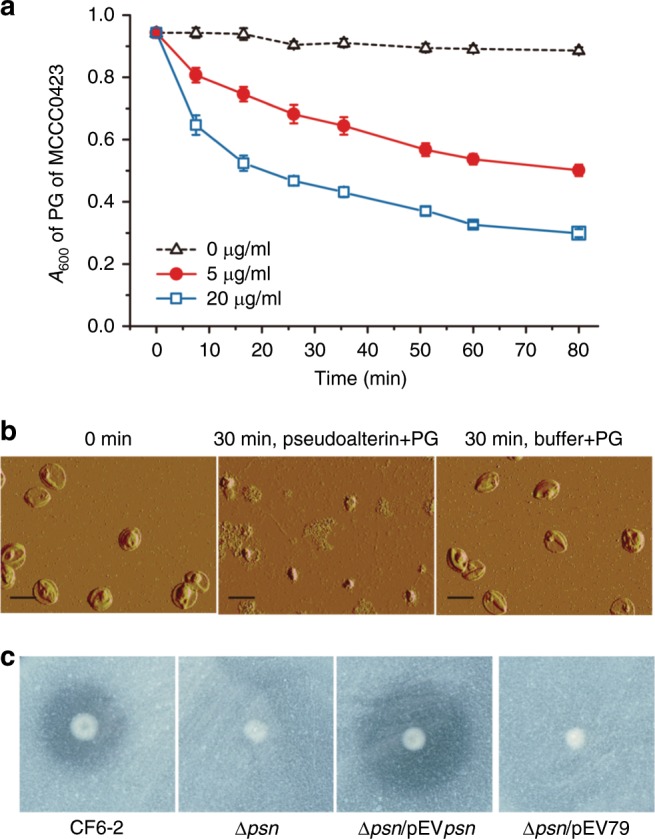
PG degradation by pseudoalterin. **a** Degradation curves of pseudoalterin towards PG of strain MCCC0423 at 25 °C. **b** AFM observation of the degradation of PG of strain MCCC0423 by pseudoalterin. Control, PG of strain MCCC0423 treated with buffer for 30 min. Bars: 1 μm. **c** PG degradation by the mutant strain Δ*psn* and the complementary strain Δ*psn*/pEV*psn* on PG-containing plates. Strains CF6-2, Δ*psn*, and Δ*psn*/pEV*psn* were spotted and grew on plates containing the PG from strain MCCC0423 at 20 °C for 3 days until clear zones formed around the colonies of strains CF6-2 and Δ*psn*/pEV*psn*. The error bar represents standard deviation of triplicate experiments. Each picture is a representative of triplicate experiments. Source data are provided as a Source Data file.

Because PG is a major cell wall component of Gram-positive bacteria, we hypothesized that pseudoalterin likely kills these Gram-positive bacteria by degrading PG. Thus, the activity of pseudoalterin against the PG from strain MCCC0423 was investigated. Indeed, pseudoalterin showed significant activity towards the degradation of purified PG from strain MCCC0423 (Fig. 3a, b). Moreover, wild-type cells of strain CF6-2 were capable of degrading PG of strain MCCC0423, whereas the Δ*psn* mutant strain had lost this ability, and this ability could be restored by complementing the *psn* gene but could not by complementing a mutant *psn* gene that encodes an inactive pseudoalterin protein (Fig. 3c). Our data therefore suggest that pseudoalterin secreted by strain CF6-2 is responsible for killing Gram-positive bacteria, likely through the degradation of PG in the cell wall although an indirect cause of cell death due to pseudoalterin treatment cannot be completely ruled out.

### Pseudoalterin production is induced by specific fragments of PG

**Fig. 4:**
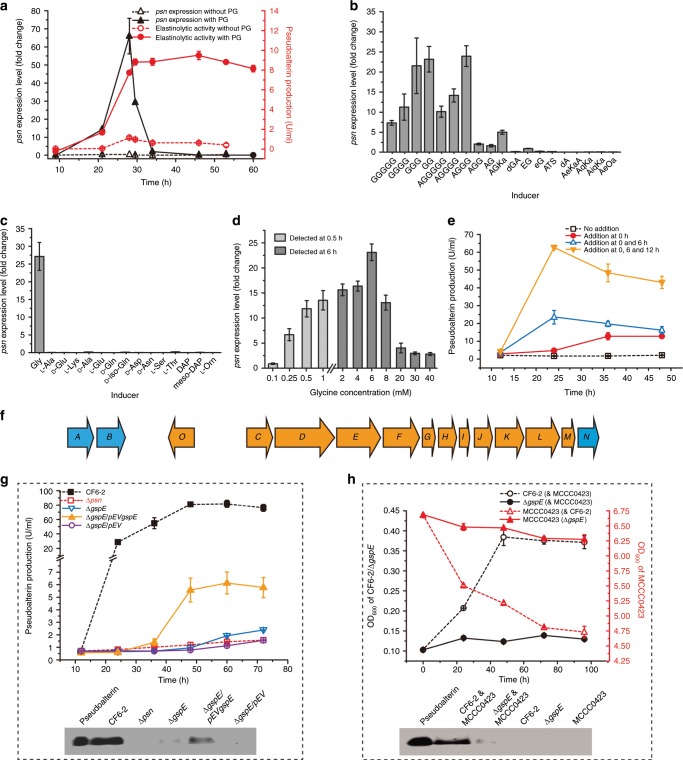
Induction and secretion of pseudoalterin in strain CF6-2. **a** Induction of *psn* expression in strain CF6-2 by PG of strain MCCC0423. Control, strain CF6-2 was cultured in the medium without PG. **b** Inducing effects of peptides derived from PG on *psn* expression. O, l-Orn; iq, d-iso-Gln. **c** Inducing effects of amino acids from PG on *psn* expression. **d** Inducing effects of different concentrations of glycine on *psn* expression. **e** The extracellular pseudoalterin production of strain CF6-2 induced by 2 mM glycine added at 0, 6, and/or 12 h. Control, strain CF6-2 was cultured in the medium without glycine. The pseudoalterin production was represented by the extracellular elastinolytic activity. **f** Genetic organization of the T2SS cluster of strain CF6-2. Each gene is represented by an arrow. The “core” genes in the cluster are marked in orange and the others (*gspA*, *B*, and *N*) in blue. **g** Pseudoalterin production of strain CF6-2 and its mutants cultured in a fermentation medium. The upper shows pseudoalterin production represented by extracellular elastinolytic activity. The lower shows pseudoalterin production detected by western blot. **h** Non-contact co-culture of strain Δ*gspE* and strain MCCC0423. The upper panel shows the growth of the strains. The lower panel shows pseudoalterin production detected by western blot. The graphs show data from triplicate experiments (mean ± SD). Source data are provided as a Source Data file.

When PG of strain MCCC0423 was used as the sole carbon and nitrogen source for strain CF6-2, pseudoalterin was expressed and secreted during the growth of strain CF6-2 cells (Fig. 4a), indicating that pseudoalterin was inducible by PG of strain MCCC0423. In the non-contact co-culture experiment, pseudoalterin production was also induced (Fig. 2a). Because PG of strain MCCC0423 is unlikely to be able to pass through the membrane, owing to its high molecular weight and low solubility, we reasoned that a soluble fragment derived from the degradation of PG of strain MCCC0423 would serve as an inducing signal molecule for pseudoalterin synthesis in strain CF6-2. To identify this signal molecule, various amino acids and peptides derived from the peptide chain of Gram-positive bacterial PG were tested for their ability for pseudoalterin induction. Both free glycine and its oligopeptides, as well as several glycine-enriching peptides including AGGGGG, AGGGG, and AGGG, could induce the transcription of *psn*, whereas other amino acids or peptides had little inducing effect (Fig. 4b, c). Of all the molecules tested, glycine had the strongest inducing effect (Fig. 4b, c). Transcription of *psn* in strain CF6-2 could be significantly induced by glycine at concentrations of 250 μM–20 mM (Fig. 4d). Moreover, 2 mM glycine in the medium induced the secretion of active pseudoalterin and the further addition of glycine after 6 and 12 h could significantly increase the production of active pseudoalterin (Fig. 4e). This indicates that the induction of pseudoalterin synthesis in strain CF6-2 needed a constant stimulation by extracellular glycine in appropriate concentrations. Glycine exists in the peptide bridges of PG of a variety of Gram-positive bacteria but absent from PG from Gram-negative bacteria and certain other Gram-positive bacteria^12^. Pseudoalterin synthesis is thus specifically induced by glycine and glycine-enriching oligopeptides derived from Gram-positive bacterial PG.

### Pseudoalterin is secreted via a type II secretion system

The type II secretion system (T2SS) of strain CF6-2 is encoded by a set of 12 *gsp* (general secretion pathway) genes, including a large operon (*gspCDEFGHIJKLMN*) and several other genes *gspA*, *gspB*, and *gspO* (Fig. 4f). A functional T2SS requires the presence of a traffic ATPase, GspE^16^. To study the secretory pathway of pseudoalterin, a mutant of strain CF6-2, Δ*gspE*, in which the gene *gspE* was knocked out, was constructed, and its ability to secrete pseudoalterin was tested. Indeed, the pseudoalterin protein and its activity were barely detectable in mutant Δ*gspE*, and a complementary mutant strain (Δ*gspE*/pEV*gspE*) partly restored pseudoalterin production and activity (Fig. 4g). In addition, the Δ*gspE* strain lost the ability to kill strain MCCC0423 cells in the non-contact co-culture (Fig. 4h). Taken together, the data suggest that pseudoalterin is secreted through T2SS.

### Mechanism of PG degradation by pseudoalterin

**Fig. 5:**
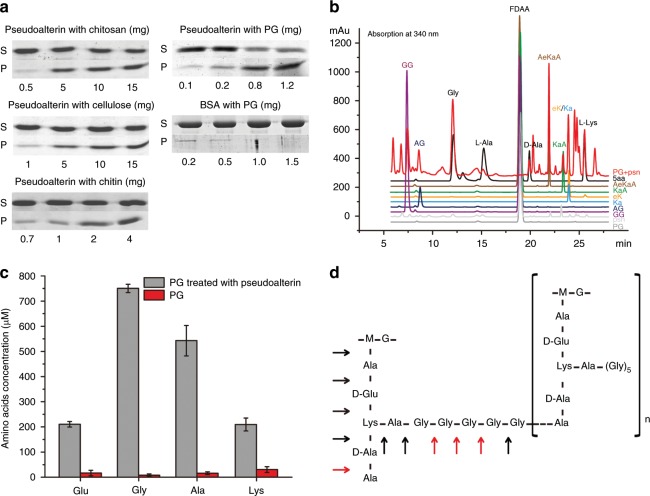
PG binding and degradation by pseudoalterin. **a** SDS-PAGE analysis of the binding ability of pseudoalterin on three insoluble polysaccharides and PG. The experiments were performed with a fixed amount of pseudoalterin (0.2 mg ml^−1^) and increasing concentrations of substrates. “S” and “P” refer to the amount of protein present in the supernatant and the pellet after centrifugation, respectively. BSA in place of pseudoalterin was used as a negative control. Each graph is a representative of at least three repeats. **b** Chiral derivatization-HPLC analysis of the d/l-amino acids and the peptides released from PG of strain MCCC0423 by pseudoalterin hydrolysis. 5aa is a mixture of five standard amino acids (Gly, d/l-Ala, d-Glu and l-Lys). **c** Analysis of the amino acids released from PG of strain MCCC0423 by pseudoalterin hydrolysis with an Automatic Amino Acid Analyzer (Hitachi L8900, Japan). Data are from triplicate experiments (mean ± SD). **d** The cleavage sites of pseudoalterin on strain MCCC0423 PG. Arrows indicate the cleavage sites determined according to analysis of the products released by pseudoalterin from two synthetic PG peptides, AaKAGGGGGA and Lactic acid-AeKAGG. Red and black arrows indicate the preferred and less preferred bonds by pseudoalterin, respectively, which are deduced based on the relative production of the released products in MS spectra (Supplementary Figs. 4 and 5). M, NAM; G, NAG. Source data are provided as a Source Data file.

Next, we set out to characterize the mechanism by which pseudoalterin degrades PG of Gram-positive bacteria using purified PG of strain MCCC0423. During the purification process of pseudoalterin, we noticed that pseudoalterin could bind to Sephadex gel matrix. This suggests that pseudoalterin may have affinity to carbohydrate chains because Sephadex gel is primarily composed of carbohydrate polymers. To test this hypothesis, the binding ability of pseudoalterin to three representative insoluble natural polysaccharides (chitosan, cellulose, and chitin) and PG was tested. Pseudoalterin could bind to all three natural polymers tested, with the highest binding ability to PG (Fig. 5a), suggesting that pseudoalterin may bind on the glycan strand of PG.

Because pseudoalterin is a member of the M23 metalloprotease family, we hypothesized that it degrades PG by attacking the peptide chain rather than the glycan strand. To test this hypothesis, the products released from the degradation of PG derived from strain MCCC0423 by pseudoalterin hydrolysis were analyzed. Chiral derivatization-HPLC analysis showed that amino acids including Gly, l-Ala, d-Ala, and l-Lys and peptides such as GG, AG, AeKaA, KaA, and eK/Ka (lowercase letter refers to the d-enantiomer of an amino acid) were released from the PG degradation (Fig. 5b). In addition, d-Glu was also detected using an Automatic Amino Acid Analyzer (Fig. 5c). These data suggest that pseudoalterin may act on the peptide chain of PG with multiple cleavage sites. Alternatively, multiple products from PG degradation could be down to the heterogeneity of the substrates (i.e. PG purified from strain MCCC0423). To determine the cleavage sites of pseudoalterin on the peptide chain of PG, two peptides, AaKAGGGGGA and Lactic acid-AeKAGG, were synthesized, based on the peptide chain sequence of *S. aureus* PG^17^. The degradation products by pseudoalterin were analyzed by LC-MS. By quantifying the relative abundance of oligopeptides released from the two synthetic peptides (Supplementary Figs. 4 and 5), we concluded that although pseudoalterin can attack all peptide, it did not cleave the peptide bonds with equal efficiency. Pseudoalterin preferred to cleave the bonds with Gly/Ala at P1 and P1′ positions and, to a lesser extent, to the bonds with larger amino acids at these positions (Fig. 5d). Since the degradation of synthetic peptides yielded a small set of preferred products (Fig. 5d), it suggests that the heterogeneous products seen in Fig. 5b emanate from the mixed population of PG substrates in that experiment. The hydrolysis of the peptide chain of PG by pseudoalterin will lead to the collapse of the cell wall and the death of the Gram-positive bacteria. The substances released from the predated cells, together with the free amino acids and oligopeptides released from PG, provide nutrients for strain CF6-2 to thrive, as observed in the co-culture of strains CF6-2 and MCCC0423 (Fig. 1b, d).

**Fig. 6:**
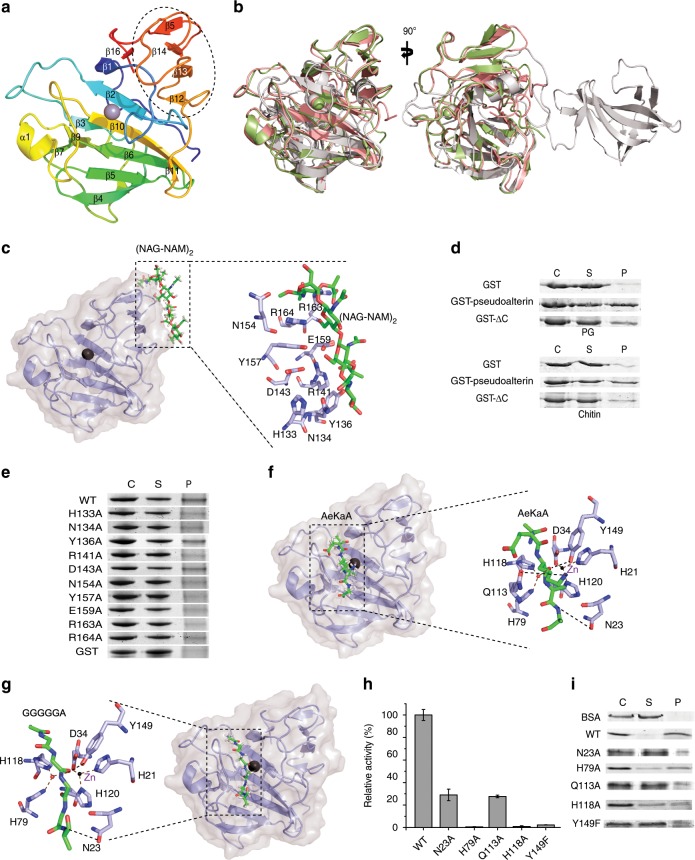
Structural basis of PG binding and degradation by pseudoalterin. **a** The overall structure of pseudoalterin. The C-terminal domain is marked in a black dotted box. **b** Structural superposition of pseudoalterin with LasA and lysostaphin. On the left is the front view and on the right is the side view. Pseudoalterin structure is colored in green, LasA in pink and lysostaphin in gray. **c** Model of the pseudoalterin:(NAG–NAM)_2_ binary complex constructed by MSD. The interface of pseudoalterin with (NAG–NAM)_2_ in the black dotted box is amplified. **e** SDS-PAGE analysis of the binding ability of GST-pseudoalterin and its site-directed mutants to PG. **d** SDS-PAGE analysis of the binding ability of GST-pseudoalterin and its C-terminal domain-deleted mutant (ΔC, N134-R173) to PG and chitin. **f** Model of the pseudoalterin:AeKaA binary complex constructed by MSD. The interface of pseudoalterin with AeKaA in the black dotted box is amplified. **g** Model of the pseudoalterin:GGGGGA binary complex constructed by MSD. The interface of pseudoalterin with GGGGGA in the black dotted box is amplified. **h** Activities of pseudoalterin and its mutants to PG. Values are normalized against the activity of WT pseudoalterin. Data are from triplicate experiments (mean ± SD). **i** SDS-PAGE analysis of the PG-binding ability of pseudoalterin and its mutants. In **d**, **e** and **i**, the experiments were performed with a fixed amount of protein (0.1 mg ml^−1^ in **d** and **e**, 0.05 mg ml^−1^ in **i**) and substrates (2.0 mg). “C” represents the total amount of protein. “S” and “P” refer to the amount of protein present in the supernatant and the pellet after centrifugation, respectively. Each graph is a representative of at least three repeats. Source data are provided as a Source Data file.

To better understand the molecular interaction between pseudoalterin and PG, the crystal structure of pseudoalterin was determined to 1.9 Å (Table 1). The sequence identity of pseudoalterin to the M23 peptidases LasA and lysostaphin is 54% and 15%, respectively. Like LasA^18^ and lysostaphin^19^, pseudoalterin contains a catalytic domain and a C-terminal domain (Fig. 6a). The catalytic domain of pseudoalterin, comprising five β-strands and four connecting loops (loop 1–loop 4), is quite similar to those of LasA and lysostaphin, with a root mean square deviation (RMSD) of 0.64 and 4.0 Å, respectively (Fig. 6a, b). Similar to that of LasA, the C-terminal domain of pseudoalterin consists of four antiparallel β-strands, and is different from that of lysostaphin that consists of eight β-strands^19^ (Fig. 6b). While the function of the C-terminal domain of LasA remains unclear, the C-terminal domain of lysostaphin interacts directly with the peptide bridge present in PG^20^.

**Table 1:** Diffraction data and refinement statistics of pseudoalterin.

	Pseudoalterin
**Data collection**	
Space group	C2221
Cell dimensions	
* a*, *b*, *c* (Å)	62.841, 82.443, 73.419
*α*, *β*, *γ* (°)	90.000, 90.000, 90.000
Resolution (Å)	50–1.9 (1.968–1.9)
*R*_merge_	0.11 (0.43)
*I* / *σI*	29.49 (3.72)
Completeness (%)	99.9 (100)
Redundancy	7.1 (7.2)
**Refinement**	
Resolution (Å)	41.314–1.9 (1.968–1.9)
No. of reflections	15331 (1484)
*R*_work_/*R*_free_	0.1538/0.1722
No. of atoms	
Protein	1382
Ligands	7
Water	170
*B*-factors	
Protein	17.74
Ligands	29.24
Water	30.84
R.m.s. deviations	
Bond lengths (Å)	0.006
Bond angles (°)	0.89

^a^Values in parentheses are for highest-resolution shell

As shown in Fig. 5a, pseudoalterin may bind on the glycan strand of PG. Since the catalytic domain is responsible for the hydrolysis of the PG peptide chain, we reasoned that the C-terminal domain is responsible for binding to the PG glycan strands. To support this hypothesis, attempts were made to model pseudoalterin with a PG fragment containing both the glycan strand and the peptide chain. Unfortunately, we failed to obtain a model with good quality. Alternatively, we modeled pseudoalterin with the glycan strand and with the peptide chain, respectively, by molecular docking simulations (MDS). In the model of the pseudoalterin: (NAG–NAM)_2_ binary complex (NAG, *N-*acetylglucosamine; NAM, *N-*acetylmuramic acid), the glycan strand (NAG–NAM)_2_ interacts with the C-terminal domain (Fig. 6c). This was supported by an analysis of the binding ability of the C-terminal domain-deleted mutant (ΔC, N134-R173) to chitin and PG. The deletion mutant lost almost all chitin-binding ability and a large portion of the PG-binding ability (Fig. 6d). Analysis of the model indicated that (NAG–NAM)_2_ interacts with the C-terminal domain mainly by forming H-bonds with the residues H133, N134, Y136, R141, D143, N154, Y157, E159, R163, and R164 (Fig. 6c). Site-directed mutations on these residues showed that the PG-binding ability of the eight mutants, H133A, N134A, R141A, N154A, Y157A, E159A, R163A, and R164A, was reduced (Fig. 6e), but the circular dichroism spectra of these mutants had no detectable differences from that of pseudoalterin (Supplementary Fig. 6). These data indicate that these residues in the C-terminal domain are involved in the binding of pseudoalterin to the PG glycan strand.

In the modeled pseudoalterin:AeKaA/GGGGGA binary complexes, AeKaA/GGGGGA is bound in the catalytic cavity of pseudoalterin with a similar mode to the tetraglycine phosphinate in LytM^21^ (Fig. 6f, g), a member of the M23 proteases that shows 19.1% sequence identity to pseudoalterin. H21, D34, and H120 chelating the zinc ion in pseudoalterin are equivalent to H210, D214, and H293 that chelate the zinc ion in LytM^21^ (Fig. 6f, g). Y204, H260, H291, N303 in LytM are involved in substrate recognition and catalysis^21^. The equivalents of these residues in pseudoalterin are Y149, H79, H118, and N23 (Fig. 6f, g). Among them, either H79 or H118 can act as a general base/acid in PG hydrolysis based on sequence alignment of pseudoalterin with its homologs (LasA, LytM, lysostaphin, and ALE-1) (Supplementary Fig. 7), and mutation of these two residues to alanine almost completely abolished pseudoalterin activity to PG (Fig. 6h). In addition, in the complexes, AeKaA/GGGGGA interacts with the residues N23, Q113, and Y14, mainly through H-bond formation (Fig. 6f, g), and mutation of these residues (N23A, Q113A, and Y149F) caused significant reduction in both the activity and the binding ability of the enzyme to PG (Fig. 6h). This suggests that these residues are involved in binding the peptide substrate. The circular dichroism spectra of all of the aforementioned mutants were indistinguishable from that of the wild-type pseudoalterin (Supplementary Fig. 8), indicating that changes in the PG binding and hydrolysis of these mutants are caused by amino acid replacement rather than structural alteration of the enzyme.

To probe the structural basis for the specificity of pseudoalterin on PG (Fig. 5c), we compared the catalytic cavity of pseudoalterin with those of LasA (PDB code: 3IT7), LytM (PDB code: 4YZB), and lysostaphin (PDB code: 4QP5) (Supplementary Fig. 9a). The catalytic cavity of pseudoalterin is similar to those of LasA and LytM, and more open than that of lysostaphin (Supplementary Fig. 9a, b), which suggests that, like these homologs, pseudoalterin also prefers to bind Gly/Ala. This is supported by our biochemical analysis presented in Fig. 5d. In addition, compared to LasA, the side chain of Q113 of loop 3 of pseudoalterin makes the S1 pocket much deeper (Supplementary Fig. 9c, d), and may be capable of orienting the long side chain of amino acids. Thus, in addition to Gly and Ala, the deeper and more open catalytic cavity of pseudoalterin may accommodate residues with a longer side chain such as Lys and Glu in PG. This makes it possible for pseudoalterin to repeatedly digest the heterogeneous PG from strain MCCC0423, generating a variety of products from PG degradation as shown in Fig. 4b, c.

Taken together, our biochemical results and structural analyses suggest that, compared to other M23 proteases, pseudoalterin has a specific conformation to bind and degrade Gram-positive bacterial PG efficiently.

### Strain CF6-2 can utilize d-Ala and d-Glu for growth

**Fig. 7:**
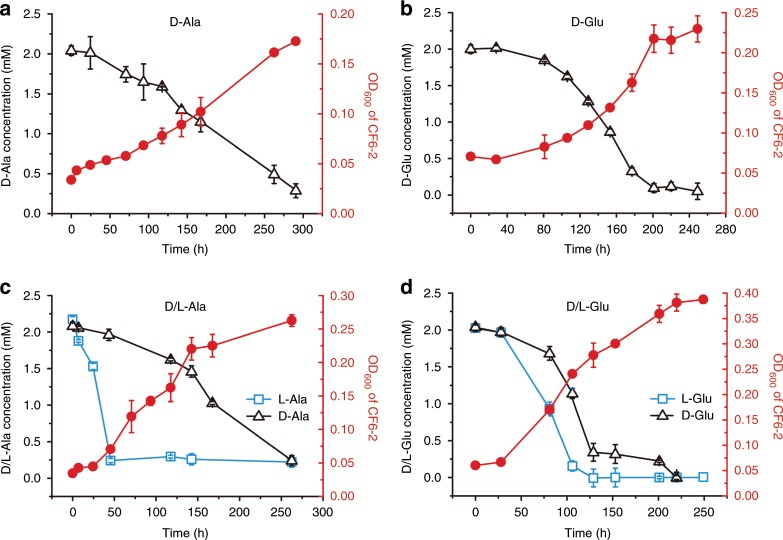
Utilization of d-Ala and d-Glu by strain CF6-2. **a** Growth curve of strain CF6-2 cultured in the medium containing 2 mM d-Ala. **b** Growth curve of strain CF6-2 cultured in the medium containing 2 mM d-Glu. **c** Growth curve of strain CF6-2 cultured in the medium containing 2 mM d-Ala and 2 mM l-Ala. **d** Growth curve of strain CF6-2 cultured in the medium containing 2 mM d-Glu and 2 mM l-Glu. The red line shows the optical density of bacterial culture measured at 600 nm and the black and blue lines indicate the concentrations of d-amino acid and l-amino acids, respectively, in the media over time. The error bars represent standard deviations from triplicate experiments. Source data are provided as a Source Data file.

As shown in Fig. 4a, wild-type strain CF6-2 can grow with PG as the sole carbon and nitrogen source, suggesting that it may be able to utilize the amino acids released from PG degradation for growth. The released amino acids include both l- and d-amino acids (Fig. 5b, c). So far, only a few bacteria are known to be able to utilize d-amino acids for growth^22^ and the recycling of d-amino acids in the marine microbial loop is not well understood. Here, the ability of strain CF6-2 to utilize the two d-amino acids, d-Ala and d-Glu, released from PG degradation was investigated. The results showed that strain CF6-2 could indeed grow on d-Ala and d-Glu as the sole nitrogen source (Fig. 7a, b). When both d- and l-Ala/Glu were present in the medium, l-amino acids were preferentially used, particularly for l-Ala (Fig. 7c, d).

### Pseudoalterin-like proteases are found in other marine bacteria

In order to better understand the ecological significance of pseudoalterin-mediated killing of Gram-positive bacteria in the environment, the distribution of pseudoalterin-like protease sequences in public databases, such as the non-redundant protein database in NCBI, was investigated. Pseudoalterin-like sequences were found in 160 marine bacterial strains which were isolated from a variety of environmental samples, including seawater (70), marine sediments (72), and hydrothermal vents (18) (Supplementary Data 1). Most of these strains are Gram-negative bacteria (134/160), particularly from the genera *Pseudoalteromonas* (30), *Vibrio* (17), *Shewanella* (13), and *Aeromonas* (5) (Supplementary Fig. 10a). The predominance of pseudoalterin-like proteases in *Pseudoalteromonas* was also confirmed by probing its distribution in the Tara Oceans Microbiome database using the Ocean Gene Atlas tool^23^. Around 86% of the pseudoalterin-like proteases from the Tara Oceans metagenomes was classified as *Pseudoalteromonas* (Supplementary Fig. 10b). We also found expression of genes encoding putative pseudoalterin-like proteases in published metatranscriptomics datasets from coastal and marine sediments, using the Integrated Microbial Genomes and Microbomes (IMG/M)^24^ database. Although only partial sequences were retrieved from these metatranscriptomes due to the length of short-reads, multiple sequence alignment confirmed that the key residues involved in the co-ordination of metals and the substrate are highly conserved (Supplementary Fig. 11). Overall, our analyses suggest that pseudoalterin-like proteases are expressed by bacteria in the ocean.

## Discussion

In addition to viral lysis and protist grazing, bacterial predation also plays an important role in marine bacterial mortality^11,25,26^. *Halobacteriovorax* species, belonging to BALOs, have been reported to be marine bacterial predators. They prey on Gram-negative bacteria by entering and residing within the periplasmic space of a host bacterium where they utilize cytoplasmic nutrients of the host to support growth and replication^27,28^. Other reported marine predatory bacteria include *Pseudalteromonas piscicida*, which preys on strains of *Vibrio* and *Shewanella*^29^, and *Saprospiraceae* strains, which prey on strains of *Photobacterium* and *Vibrio*, as well as diatoms and cyanobacteria^30^.

**Fig. 8:**
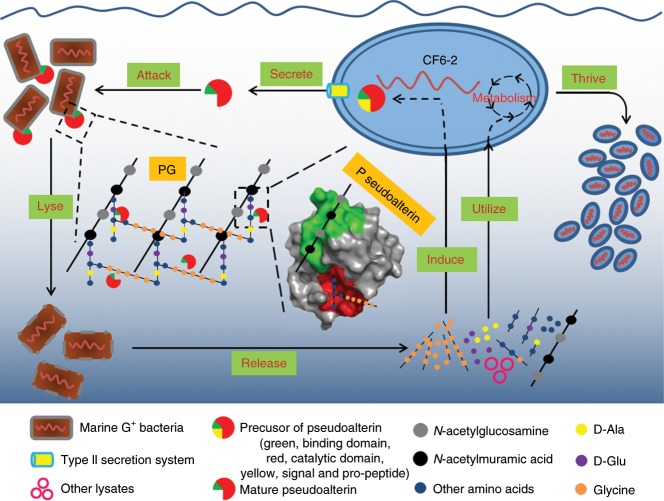
A model for Gram-negative strain CF6-2 preying on Gram-positive marine bacteria for nutrients with the secreted protease pseudoalterin as a weapon. Strain CF6-2 lyses Gram-positive bacterial cells by secreting the metalloprotease pseudoalterin to degrade the PG in Gram-positive bacterial cell wall, which leads to the collapse of the cell wall and subsequent lysis of the cell. Then, strain CF6-2 utilizes the d/l-amino acids and the oligopeptides released from PG degradation and the substances from the inside cell to thrive. In the meantime, the released glycine and glycine-enriching oligopeptides from the degradation of Gram-positive bacterial PG induce the synthesis of pseudoalterin in strain CF6-2, and then the synthesized pseudoalterin is secreted through the T2SS of strain CF6-2.

In this study, we describe a new predator–prey interaction between Gram-negative and Gram-positive bacteria in the ocean. We show that the Gram-negative bacterium strain CF6-2 isolated from deep-sea sediment can kill a variety of Gram-positive bacteria with different PG chemotypes. Unlike *Halobacteriovorax* species that are intracellular predators^27,28^, strain CF6-2 is an extracellular predator, which kills Gram-positive bacteria by secreting an extracellular protease, pseudoalterin, that degrades the PG in the prey’s cell wall (Fig. 8). Interestingly, pseudoalterin has no killing activity against Gram-negative bacteria or against Gram-positive bacteria of the *Mycobacterium* genus (Fig. 2e). Furthermore, pseudoalterin had killing activity against the Gram-positive bacterium *Pontibacillus* sp. MCCC1A04056, whereas no activity was found against Gram-positive bacteria *Mycobacterium* spp., although they all have the same PG chemotype^12,16,31,32^. The PG of the aforementioned *Mycobacterium* strains is covered by a large number of unusual lipids in the cell wall^31^ and that of Gram-negative bacteria is sandwiched between two membranes. Thus, inaccessibility of pseudoalterin to the bacterial PG may be the main reason why pseudoalterin cannot kill these bacteria. A bioinformatics investigation showed that other Gram-negative bacteria possess genes encoding putative pseudoalterin-like proteases. Further metagenome and metatranscriptome analyses showed that similar genes are found and expressed in marine environments.

There are several lines of evidence to support the conclusion that strain CF6-2 preys on some Gram-positive marine bacteria for nutrients using the secreted pseudoalterin as a weapon. (i) Pseudoalterin synthesis is only induced by characteristic fragments from Gram-positive bacterial PG, e.g. glycine and glycine-rich oligopeptides. This can explain why strain CF6-2 cannot kill Gram-negative bacteria or some Gram-positive bacteria on agar plates, because the PG of these bacteria contains no glycine and thus is unable to induce pseudoalterin synthesis. Although it has been speculated that specific fragments from PG are signaling molecules for PG hydrolase induction in predatory bacteria^13^, neither the identity of the fragments nor the bacterial receptors for these fragments have been identified. (ii) Pseudoalterin can bind to the glycan strand of Gram-positive bacterial PG with its C-terminal domain and degrade the PG peptide with its catalytic domain. Unlike other reported PG hydrolases, which usually have one or limited cleavage sites on PG^18,19,33^ pseudoalterin seems to be able to cleave all the peptide bonds in the Gram-positive bacterial PG peptide chain, although it shows preference for bonds with Gly/Ala at P1/P1′ positions. In addition, although pseudoalterin is only induced by glycine and glycine-enriching oligopeptides, it can also degrade bacterial PG without glycine.

Glycine resulting from PG degradation is a strong inducer for pseudoalterin expression in strain CF6-2 (Fig. 4). Indeed, qRT-PCR results showed that pseudoalterin is induced above background at 100 μM glycine in the medium. However, glycine concentration rarely reaches μM in surface seawater, hence the significance of pseudoalterin-mediated predation is uncertain in marine water columns. Yet, our metagenome analyses have confirmed that bacteria in seawater do have the genetic potential and *Pseudoalteromonas* represents the major clade encoding pseudoalterin-like proteins. In coastal and marine sediments, however, several reports have shown that glycine concentration in sediment pore water as well as sediment traps can reach hundreds of μM^34,35^. Coincidently, *Pseudoalteromonas* is frequently detected as an important member of the microbial community in marine sediments^36,37^. It is therefore likely that pseudoalterin-mediate predation may occur in marine sediments where the Gly concentration for inducing pseudoalterin expression can be achieved locally. Indeed, this view is further supported by the analyses of metatranscriptomics datasets, showing expression of genes encoding pseudoalterin-like proteases in sediment samples. Taken together, the data presented in this study suggest that *Pseudoalteromonas* can prey on Gram-positive bacteria by secreting pseudoalterin. Whether this strategy is adopted by marine bacteria in situ awaits further investigation.

## Methods

### Bacterial strains, growth conditions, and materials

*Pseudoalteromonas* sp. CF6-2 and *Bacillus* sp. CF12-9 were originally isolated from a marine sediment sample from the South China Sea^14^. Other bacterial strains used in this study (Supplementary Table 1) were obtained from MCCC (Marine Culture Collection of China). All strains were cultivated at 20 °C on a plate containing marine Luria-Bertani (LB) medium composed of (w/v) 1% peptone, 0.5% yeast extract, 1.5% agar and 3% sea salt (pH 8.0), and then stored at 4 °C for short-term use, or mixed with 15% glycerin (v/v) and stored at −80 °C for long-term use. Sea salt was purchased from Sigma-Aldrich (USA), and Transwell^®^ Permeable Supports (Diameter, 24 mm; pore size, 0.4 μm) from Corning (USA). PG was extracted from strain MCCC0423 cells using the method of Takano et al.^38^.

### Construction of mutants

The knockout mutants of strain CF6-2, Δ*psn* and Δ*gspE*, as well as their complementary strains, Δ*psn*/pEV*psn* and Δ*gspE*/pEV*gspE*, were constructed as described previously^39–41^. The plasmids, bacterial strains, and primers used in this study are listed in Supplementary Tables 2 and 3 and Supplementary Data 2. Site-directed mutagenesis in pseudoalterin was introduced by PCR and the mutations were verified by DNA sequencing. For recombinant protein expression, the plasmid pGEX-4T-1 containing the appropriate insert was expressed in *Escherichia coli* strain BL21 (DE3) at 15 °C, 100 r.p.m. for 10 h, with 0.1 mM isopropyl β-d-1-thiogalactopyranoside (IPTG) as an inducer. The proteins were purified with glutathione-Sepharose 4B matrix (GE Healthcare, Sweden). For the expression of active pseudoalterin and its mutants, the plasmid pEV containing the appropriate insert was expressed in strain Δ*psn*, the *psn*-deleted mutant of strain CF6-2, and the expressed proteins were purified as described previously^15^. The binding ability of the wild-type pseudoalterin and its mutants to Gram-positive bacterial PG and other polysaccharides was analyzed using the method of Valenzuela et al.^42^. The circular dichroism spectra of pseudoalterin and its mutants were monitored between 200 and 240 nm at 25 °C on a Jasco J-810 spectropolarimeter (Japan).

### Predation activity of strain CF6-2 and its mutants on bacteria

Strain CF6-2 (or its mutants) and the target bacterial strains were pre-cultured in liquid marine LB medium overnight. Cells were collected by centrifugation and re-suspended in artificial seawater. The OD_600_ of the cell suspensions was adjusted with artificial seawater according to the needs of the following experiments. To detect the killing activities of strain CF6-2 and its mutants against other bacteria on a plate, Burkholder agar diffusion assays were performed as described previously^43^. Briefly, the target bacterium layer was prepared by mixing 1% (v/v) bacterial suspension (OD_600_ ≈ 1.0) with 0.6% (w/v) molten ZoBell agar in artificial seawater. Then, 2 μl of strain CF6-2 (or its mutants) (OD_600_ ≈ 0.1) was spotted on to the agar surface. The plates were incubated face-up at 20 °C for 3 days. To test the predation activity of strain CF6-2 on strain MCCC0423 in liquid culture, 1% (v/v) cell suspensions of strain CF6-2 and strain MCCC0423 (OD_600_ ≈ 1.0) were mixed in artificial seawater and were cultivated at 20 °C with shaking. Then, the colony formation units (CFU) for each of the bacteria were calculated at regular intervals for 20 h. Individual cultures of strain CF6-2 and strain MCCC0423 in artificial seawater were used as controls. Experiments were performed in three biological replicates.

The non-contact interaction between strain CF6-2 (or the Δ*gspE* mutant) and strain MCCC0423 was performed in a Transwell^®^ permeable support device. Strain CF6-2 or the mutant ΔgspE cells (OD_600_ ≈ 0.1, 1.5 ml) were added into the upper insert. Strain MCCC0423 cells (OD_600_ ≈ 6.0, 2.6 ml) were added into the lower well. The device was then incubated at 20 °C, and the OD_600_ of the cultures in both the lower well and the upper insert was measured every 20 h. In addition, the supernatant from the non-contact co-culture was boiled for 10 min to determine the effect of heat-treatment on the lysis activity of the supernatant to the cells of strain MCCC0423. Furthermore, 2 mM of Zn^2+^ was added in the Transwell experiment to determine its impact on the non-contact interaction between strains CF6-2 and MCCC0423. All experiments were performed in three biological replicates.

### Western blot analysis

After the non-contact co-culture of strain CF6-2/Δ*gspE* and strain MCCC0423 were incubated at 20 °C for 60 h, proteins in 1 ml of the culture supernatant in the lower well was precipitated by adding 100 μl 1.25 M trichloroacetic acid. The precipitated proteins were dissolved in 10 μl of 50 mM Tris-HCl (pH 9.0). Strain CF6-2, Δ*gspE*, or Δ*gspE*/pEV*gspE*, cultured in a fermentation medium as described previously^15^ at 20 °C for 60 h, were used as controls. All samples were subjected to sodium dodecyl sulfate polyacrylamide gel electrophoresis in 15% (w/v) polyacrylamide separating gels and western blotting was performed as described previously^44^. The blots were probed for pseudoalterin using a pseudoalterin polyclonal antibody (1000× dilution of 3.4 mg ml^-1^) prepared by the Beijing Genomics Institute, China. Following treatment with a horseradish peroxidase-conjugated secondary antibody (goat anti-rabbit ab6721, Abcam, 1000× dilution of 2 mg ml^−1^), the blots were imaged in chemiluminescent solution (GE Healthcare, Amersham^TM^ ECL^TM^ Prime Western Blotting Detection Reagent, RPN2232) on a myECL™ Imager (Thermo Scientific).

### Purification and structure determination of pseudoalterin

Pseudoalterin was first purified from the strain CF6-2 culture as previously described by Zhao et al.^15^, and then further purified by gel filtration using a Sepharose 6B column (GE Healthcare) to obtain highly purified protein. The purified protein was concentrated to 2 mg ml^−1^ and subjected to crystallization immediately. Diffraction-quality crystals of pseudoalterin were harvested in hanging drops containing 0.1 M Bis–Tris (pH 7.1) and 0.6 M magnesium formate dehydrate after 7 days’ incubation at 4 °C. The crystals were soaked in the buffer containing 20% (v/v) glycerin, 0.1 M Bis–Tris (pH 7.1), and 0.6 M magnesium formate dehydrate for 20 s and then freezed in liquid nitrogen before X-ray diffraction. X-ray diffraction data were collected from single crystals at 100 K on the BL17U1 beamline at the Shanghai Synchrotron Radiation Facility (SSRF, China). The collection wavelength is 0.9791 Å. The crystal structure of pseudoalterin was solved using the structure of LasA (PDB code 3IT5) as a model. Automated model building was performed with Auto Build in PHENIX. Several rounds of refinement and manual building were then performed with phenix.refine and Coot, respectively. Data refinement statistics are given in Table 1. Ramachandran Plot analysis suggests that 97.04% of the residues are in the favored region and 2.96% of the residues are in the allowed region.

### Molecular docking simulations

MDS was conducted on Molecular Operating Environment (MOE) v2018.0101. The structure of (NAG–NAM)_2_ was adopted from PDB 4BPA. The structures of AeKaA and GGGGGA were generated by the MOE-Protein builder module. Both structures were prepared in MOE through energy minimization. For the production of modes of pseudoalterin with PG-derived peptide fragments, the ligands were treated as flexible and protein was treated as rigid body. Prior to docking, the force field of AMBER10:EHT and the implicit solvation model of Reaction Field were selected. MOE-Dock was used for MDSs of molecules with proteins. The docking workflow followed the “induced fit” protocol, in which the side chains of the receptor pocket were allowed to move according to ligand conformations, with a constraint on their positions. The weight used for tethering side chain atoms to their original positions was 10. For each ligand, 1000 docked poses were ranked by London dG scoring first, and then a force field refinement was carried out on the top 50 poses followed by a rescoring of GBVI/WSA dG. The conformations with the lowest free energies of binding were selected as the best (probable) binding modes. Molecular graphics were generated by PyMOL.

### Pseudoalterin activity assays

The activity assays of pseudoalterin towards elastin and PG were carried out according to Zhao et al.^15^ and Iversen et al.^45^, respectively. One unit of activity towards elastin was defined as the amount of enzyme that is required for an increase of 0.01 unit of absorbance at 590 nm per min. One unit of activity towards PG was defined as the amount of enzyme that is required for an decrease of 0.01 unit of absorbance at 600 nm per min.

The lytic activity of pseudoalterin to live cells or purified PG was assayed using the method reported by Sugai et al.^46^, with minor modification. Briefly, bacterial cells were harvested in the exponential growth phase. The cells or purified PG were re-suspended in 20 mM Tris-HCl (pH 9.0) and the OD_600_ was adjusted to 0.8–1.0. Bacterial cells were mixed with 0.2 or 1.0 μg ml^−1^ pseudoalterin, and PG with 5 or 20 μg ml^−1^ pseudoalterin. The mixtures were incubated at 25 °C, and the OD_600_ values of the mixtures were measured every 10 min for strain MCCC0423 cells and PG or after 120 min for other bacteria.

### SEM and TEM

The cells of strain MCCC0423 (OD_600_ ≈ 1.0) were mixed with 0.1 μg ml^−1^ pseudoalterin in 20 mM Tris-HCl (pH 9.0) at 25 °C for 30 min (SEM) or 1 h (TEM), and then were fixed in 2.5% glutaraldehyde for at least 2 h. After fixation, the samples were washed three times with distilled water. For SEM, the fixed samples were then dehydrated by ethanol solutions of 60%, 70%, 80%, 90%, and 100% (for two times) for 15 min each. Afterwards, the samples were dried with a critical point drier (LEICA EM CPD300, Germany), gold sputtered (CRESSINTON SPUTTER COATER 108, UK) and examined with an Environmental Scanning Electron Microscope (FET QUANTA FEG 250, USA).

For TEM, the fixed samples were further fixed with 2% osmium tetroxide for 2 h, and then washed three times with 20 mM Tris-HCl (pH 9.0). The fixed samples were dehydrated by ethanol solutions of 30%, 50%, 70%, 90%, 95%, and 100% (two times) for 10 min each. After dehydration, the ethanol solutions were substituted by acetone, and epoxy resin infiltration was done (step 1: acetone:epoxy resin = 3:1, 2 h; step 2: acetone:epoxy resin = 1:1, 2 h; step 3: acetone:epoxy resin = 2:3, overnight; step 4: 100% epoxy resin, 24 h). After that, 2% catalytic agent was added into the epoxy resin and the epoxy resin that contains the samples was polymerized after heating and thermal insulation (step 1: 35 °C, 12 h; step 2: 45 °C, 12 h; step 3: 60 °C, 24 h). Then the samples were cut into ultrathin sections by a ultramicrotome (Leica EM UC6, Germany). The ultrathin sections were stained with both uranyl acetate (1%, 30 min) and lead citrate (1%, 5 min) and were observed by a transmission electron microscope (JEM1200-EX, Japan).

### Atomic force microscopy

AFM was carried out using a Multimode VIII AFM with Nanoscope V controller (Bruker AXS, Germany) equipped with a J-type scanner. To observe the PG degradation by pseudoalterin, PG of strain MCCC0423 (OD_600_ ≈ 0.8–1.0) was mixed with 5.0 μg ml^−1^ pseudoalterin, and then incubated at 25 °C for 30 min with stirring. For PG imaging, a drop (1.5 μl) of PG suspension was deposited onto freshly cleaved mica, and kept at ambient temperature for drying. Then the AFM imaging was carried out in scanasyst mode in air condition. Silicon cantilevers (XSC_11_/ALBS, MikroMash, Bulgaria) with a spring constant of approximately 2.7 nm^−1^ were used for imaging^47^. For imaging the lysis process of strain MCCC0423 cells by pseudoalterin, freshly cleaved mica was first modified with poly-l-lysine. Then a drop (10 μl) of strain MCCC0423 cells suspension was deposited onto mica and incubated for 5 min in a humidor to allow an adsorption of cells onto the surface. The surface was then rinsed to remove bacterial cells that were not adsorbed to the substrate. The sample was mounted onto the AFM liquid cell, and a solution of pseudoalterin (2.0 μg ml^−1^) was injected into the AFM liquid cell. Continuous imaging of strain MCCC0423 cells was carried out in contact mode in liquid condition with silicon nitride cantilevers (NP-S10, Bruker) with a spring constant about 0.32 N m^−1^. Image processing and analysis were performed with AFM off-line software NanoScope Analysis (Bruker AXS, Germany).

### Hydrolytic products and cleavage sites of pseudoalterin on PG

PG (OD_600_ ≈ 1.0) was digested with 20 μg ml^−1^ pseudoalterin in 20 mM Tris-HCl (pH 9.0) at 25 °C for 24 h. The released amino acids in the hydrolysate were analyzed by an Automatic Amino Acid Analyzer (Hitachi L8900, Japan). The d/l-amino acids and peptides in the hydrolysate were also analyzed by HPLC. The d/l-amino acids in the hydrolysate were derivatized with FDAA (*Nα*-(2,4-dinitro-5-fluorophenyl)-l-alaninamide, Marfey’s reagent; Sigma) as described by Hess et al.^48^. Derivatized amino acids were separated with a linear gradient of formic acid (30 mM, pH 2.6)/acetonitrile on an HPLC with an Inertsil ODS-3 column (250 × 4.6 mm; 5 μm particle size) (Shimadzu, Japan) at a flow rate of 1.5 ml min^−1^ and detected at 340 nm. Glycine, d-alanine, l-alanine, l-lysine, d-glutamate, AG, AGG, AGGG, AGGGG, AGGGGG, GG, GGG, GGGG, GGGGG, Ae, AeK, AeKa, AeKaA, eK, eKa, eKaA, Ka, KaA, and aA at concentrations of 0.5–4.0 mg ml^−1^ served as standard solutions.

Peptides AaKAGGGGGA and lactic acid-AeKAGG were synthesized by ChinaPeptides Co., Ltd (China). Each of these peptides (50 μg) was hydrolyzed using 2.5 μg pseudoalterin in 25 μl of 10 mM Tris-HCl (pH 9.0) at 20 °C for 25 h. The hydrolysates were subjected to LC-MS analysis to determine the molecular masses of the released peptides. The sequences of the released peptides were determined by using ExPASy tools.

### Expression and production of pseudoalterin of strain CF6-2

The expression level of pseudoalterin in strain CF6-2 was detected by real-time quantitative PCR (qPCR). Strain CF6-2 was incubated at 20 °C in artificial seawater containing 0.8% (w/v) PG of strain MCCC4032, 1% (w/v) mannitol and 2 mM peptides, 1% (w/v) mannitol and 2 mM amino acid, or 1% (w/v) mannitol and different concentrations of glycine. Artificial seawater containing 1% (w/v) mannitol and 0.5% (w/v) casamino acid served as the negative control. All of these media contained 0.5 mM CaCl_2_ and 0.5 mM Na_2_HPO_4_. Total RNA was extracted with the RNeasy Mini Kit (Qiagen, Valencia, CA, USA). Reverse transcription was performed using the PrimeScriptTM RT reagent Kit with gDNA Eraser (Perfect Real Time) (TaKaRa, Japan). The qPCR reaction was performed on the LightCycler^®^ 480 (Roche, Switzerland). The relative expression level was indicated as fold change which was calculated using the LightCycler^®^ 480 software. Each sample for qPCR was performed in triplicate and a mean value and standard deviation were calculated. Primers used for qPCR are listed in Supplementary Data 2.

Pseudoalterin production of strain CF6-2 induced by glycine was determined by measuring the extracellular elastinolytic activity as described previously^15^. Strain CF6-2 was incubated at 20 °C in artificial seawater containing (w/v) 0.2% yeast extract, 0.5% casein, 0.5 mM CaCl_2_, and 0.5 mM Na_2_HPO_4_. As an inducer, 2 mM glycine was added into the medium at 0, 6, and/or 12 h. The extracellular elastinolytic activity was measured every 12 h. Strain CF6-2 was incubated in the medium without glycine served as the negative control.

### Utilization of d-Ala and d-Glu by strain CF6-2

Strain CF6-2 was incubated at 20 °C in the media with amino acids as the sole nitrogen source. The media contained 50 mM glucose, 3.0% (w/v) synthetic sea salt (Sigma-Aldrich, USA), 0.2 M phosphate buffer (pH 8.0), and 2 mM d-amino acid (or a mixture of 2 mM d-amino acid and 2 mM l-amino acid). The OD_600_ of the cultures was measured at different time points to detect the growth of strain CF6-2. The concentrations of d- or l-amino acid in the cultures were determined by using circular dichroism (Jasco J-810 Spectropolarimeter, Japan)^49^ or by using an Automatic Amino Acid Analyzer (Hitachi L8900, Japan).

### Bioinformatics

The NCBI non-redundant protein database was used to search for the distribution of pseudoalterin-like proteases in genome-sequenced bacterial isolates (Supplementary Data 1). Pseudoalterin amino acid sequence of strain CF6-2 (accession number ADU33224) was used as the query with an *e*-value cut-off of *e*^−5^. The Tara Oceans Microbiome (Reference Gene Catalog V1, prokaryotes) was probed for the distribution of pseudoalterin-like proteases in marine metagenomes using ADU33224 as the query sequence with an *e*-value cut-off of *e*^−20^, which resulted in 165 hits. Phylogenetic distribution of these homologs retrieved from the Tara Oceans metagenome was analyzed using the Krona toolset^50^ and the data are presented in Supplementary Fig. 10. The IMG/M metatranscriptome database was used to search for the transcription of pseudoalterin-like proteases in natural coastal and marine sediments (Supplementary Data 3). The Pseudoalterin sequence of strain CF6-2 (ADU33224) was used as the query with an *e*-value cut-off of −20.

### Reporting summary

Further information on research design is available in the Nature Research Reporting Summary linked to this article.

## Supplementary information

Supplementary Information

Description of Additional Supplementary Files

Supplementary Movie 1

Supplementary Data 1

Supplementary Data 2

Supplementary Data 3

Reporting Summary

## Data Availability

The structure of pseudoalterin has been deposited in the Protein Data Bank (PDB) under the accession code 6IK4.

## Source data

Source Data
