# Supplementary material for: A predator-prey interaction between a marine Pseudoalteromonas sp. and Gram-positive bacteria
Source: Nat Commun. 2020 Jan 15;11:285. doi: 10.1038/s41467-019-14133-x (PMC6962226; doi:10.1038/s41467-019-14133-x)
Supplement: Supplementary file 1 — Supplementary Information [file 41467_2019_14133_MOESM1_ESM.pdf]

Supplementary Information

A predator-prey interaction between a marine *Pseudoalteromonas* sp. and Gram-positive bacteria

Tang et al

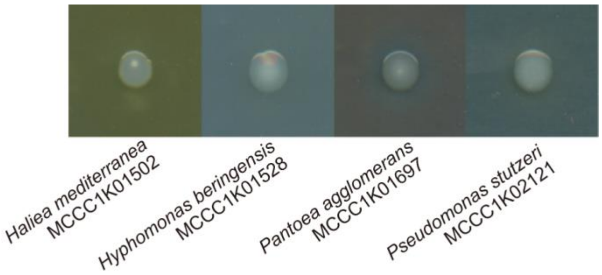

Supplementary Figure 1 | Interaction of strain CF6-2 with Gram-negative marine bacteria on agar plates. Strain CF6-2 was spotted on to the surface of the agar layer containing a Gram-negative bacterial strain, and the plates were incubated face-up at 20°C for 3 days.

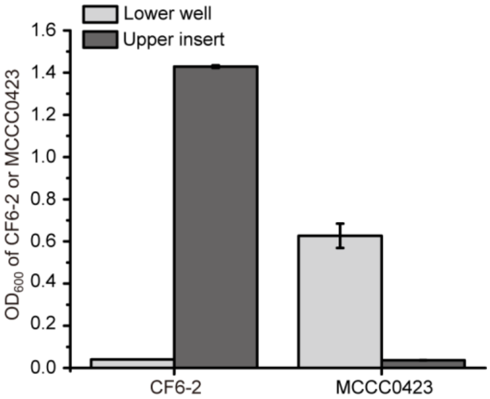

Supplementary Figure 2 | The growth of strain CF6-2 or strain MCCC0423 cultured alone in the Transwell® permeable supports device. When strain CF6-2 was cultured, 1% strain CF6-2 cell suspension (OD_{600}≈1.0) was added in the upper insert and marine LB medium was added in the lower cell. When strain MCCC0423 was cultured, 1% strain MCCC0423 cell suspension (OD_{600}≈1.0) was added in the lower cell and marine LB medium was added in the upper insert. The OD_{600} of the cultures in both the lower well and the upper insert was measured after 24 h incubation at 20°C. The result showed that strain CF6-2 or strain MCCC0423 cells could not pass the permeable membrane at the inset bottom. The error bar represents standard deviation from triplicate experiments. Source data are provided as a Source Data file.

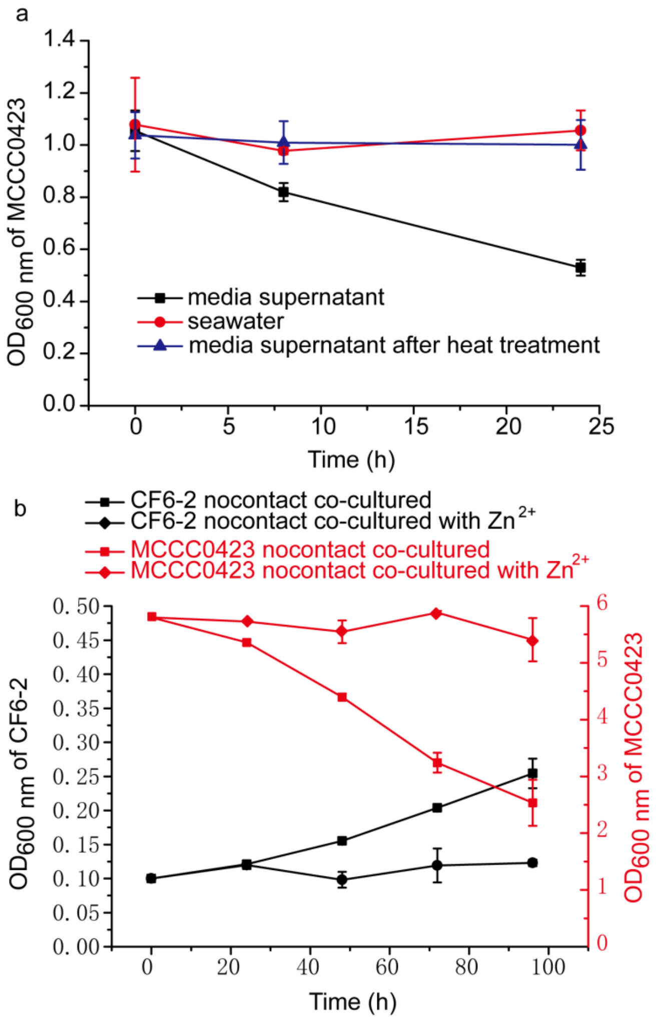

Supplementary Figure 3 | Effects of heat-treatment (a) and protease inhibitor (b) on the cell killing activity of the compound(s) secreted by strain CF6-2 in the non-contact co-culture of strains CF6-2 and MCCC0423. a, The supernatant from the non-contact co-culture was boiled for 10 min to detect the effect of heat-treatment on the cell killing activity of the compound(s) in the supernatant to strain MCCC0423. b, Zn^{2+} at concentration of 2 mM was added in the Transwell experiment to detect the effect of protease inhibitor on the non-contact interaction between strains CF6-2 and MCCC0423. The error bar represents standard deviation from triplicate experiments. Source data are provided as a Source Data file.

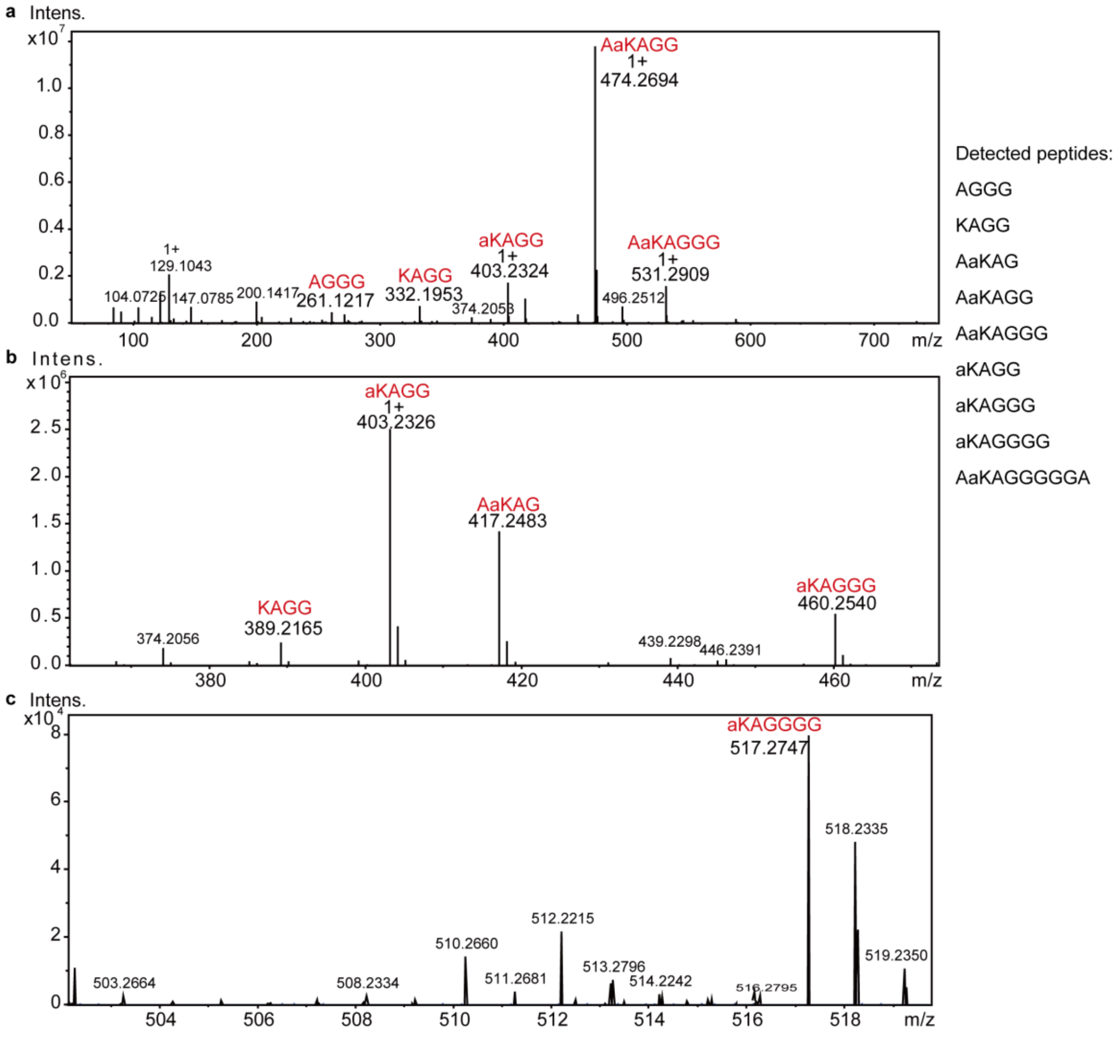

Supplementary Figure 4 | Analysis of the molecular masses and sequences of the peptides released from the peptide AaKAGGGGGA by pseudoalterin. a, sequences at 10^7 intensity level. b, sequences at 10^6 intensity level. c, sequences at 10^4 intensity level. AaKAGGGGGA was synthesized according to the sequence of Gram-positive bacterial PG peptide chain. Molecular masses were determined by LC-MS, and sequences were determined by using ExPASy tools.

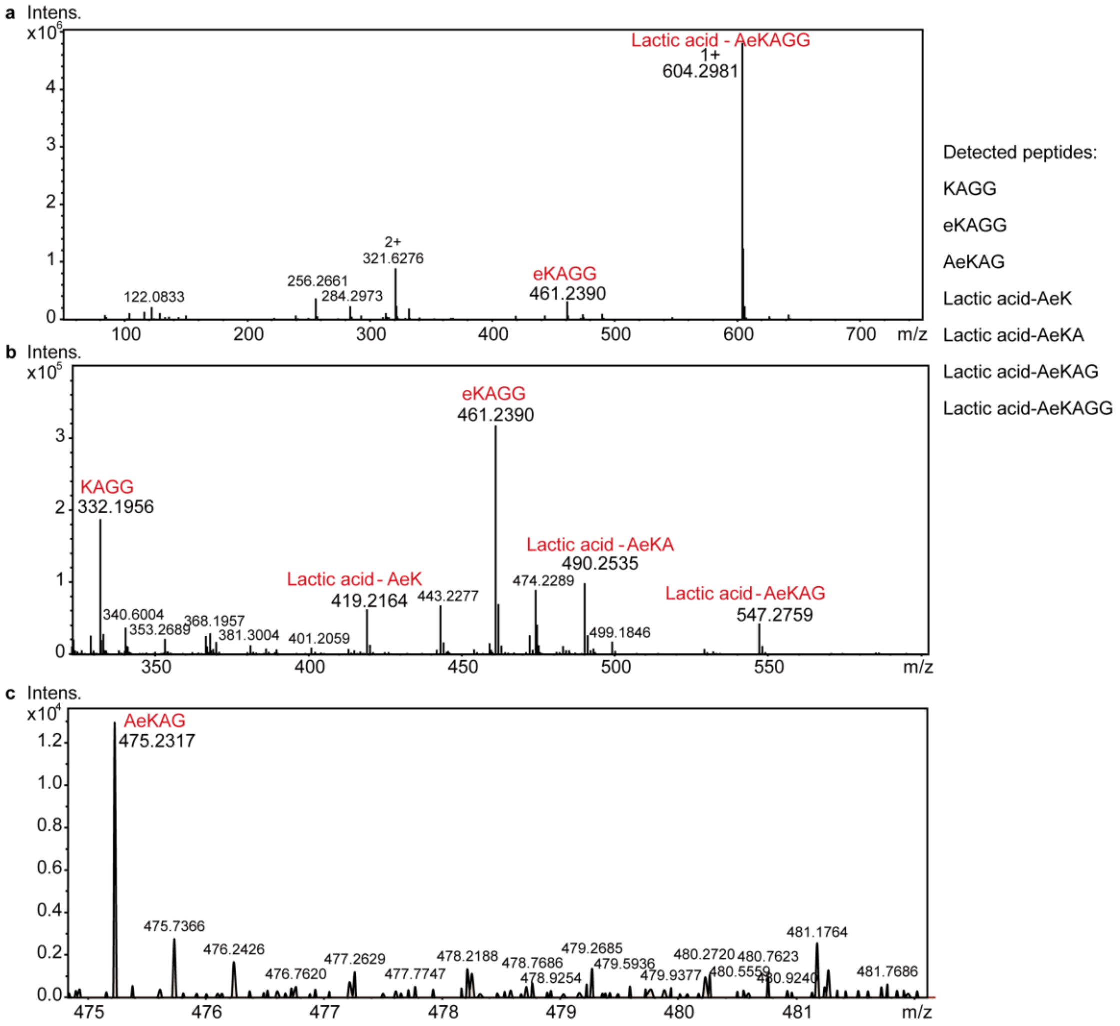

Supplementary Figure 5 | Analysis of the molecular masses and sequences of the peptides released from the peptide Lactic acid-AeKAGG by pseudoalterin. a, sequences at 10^6 intensity level. b, sequences at 10^5 intensity level. c, sequences at 10^4 intensity level. Lactic acid-AeKAGG was synthesized according to the sequence of Gram-positive bacterial PG peptide chain. Molecular masses were determined by LC-MS, and sequences were determined by using ExPASy tools.

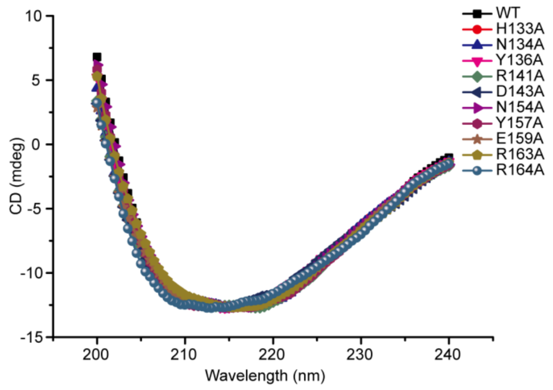

Supplementary Figure 6 | The circular dichroism spectra of GST-pseudoalterin and its mutants. The GST-pseudoalterin and its mutants expressed in *Eschericha coli* contain a GST tag and the whole sequence (403 amino acid residues) of pseudolterin precursor.

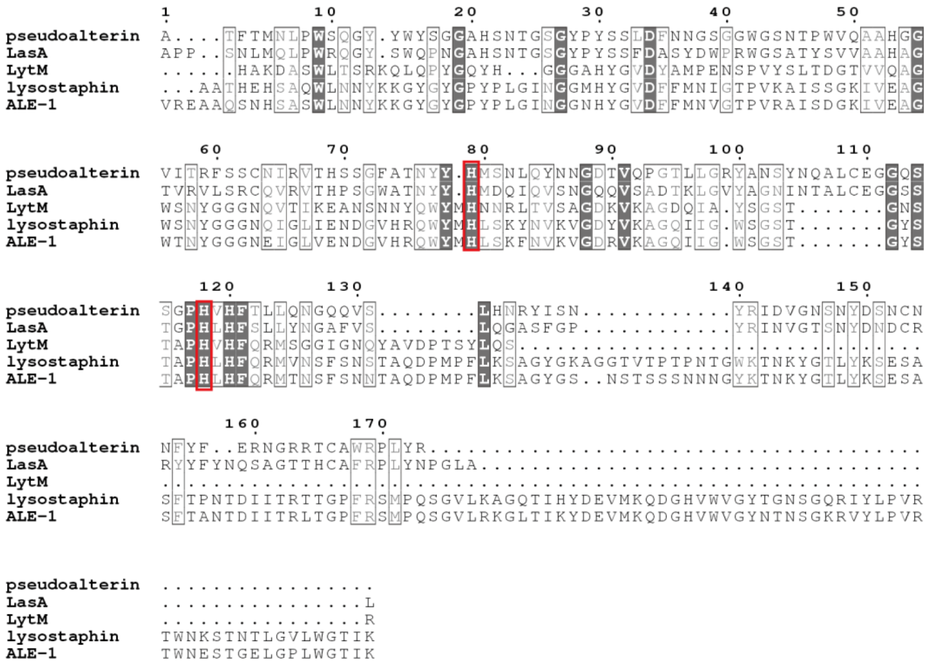

Supplementary Figure 7 | Sequence alignment of pseudoalterin with other M23 proteases. Other M23 proteases include LasA (WP_031768536.1) from *Pseudomonas aeruginosa*, LytM (2B0P_A) from *Staphylococcus aureus*, Lysostaphin (WP_013012297.1) from *Staphylococcus simulans*, and ALE-1 (O05156.1) from *Staphylococcus capitis*. Numbering is according to the sequence of mature pseudoalterin. Identical amino acids are shaded in black. Similar amino acids are framed by gray box. Identical amino acids that may act as a general base/acid in catalysis are

framed by red box.

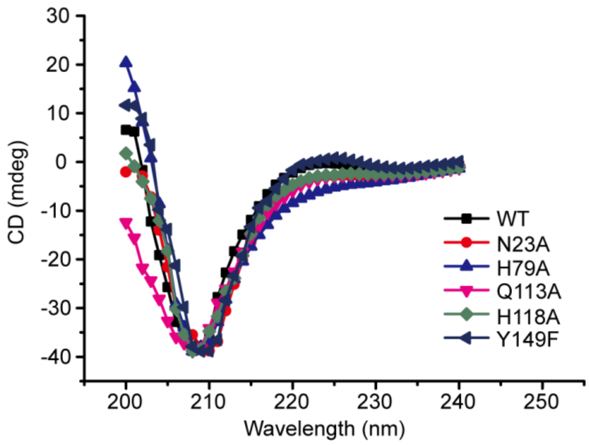

Supplementary Figure 8 | The circular dichroism spectra of pseudoalterin and its mutants. Pseudolterin and its mutants expressed in the mutant *Δpsn* of strain CF6-2 contain a His tag and the sequence (173 amino acid residues) of the active pseudolterin.

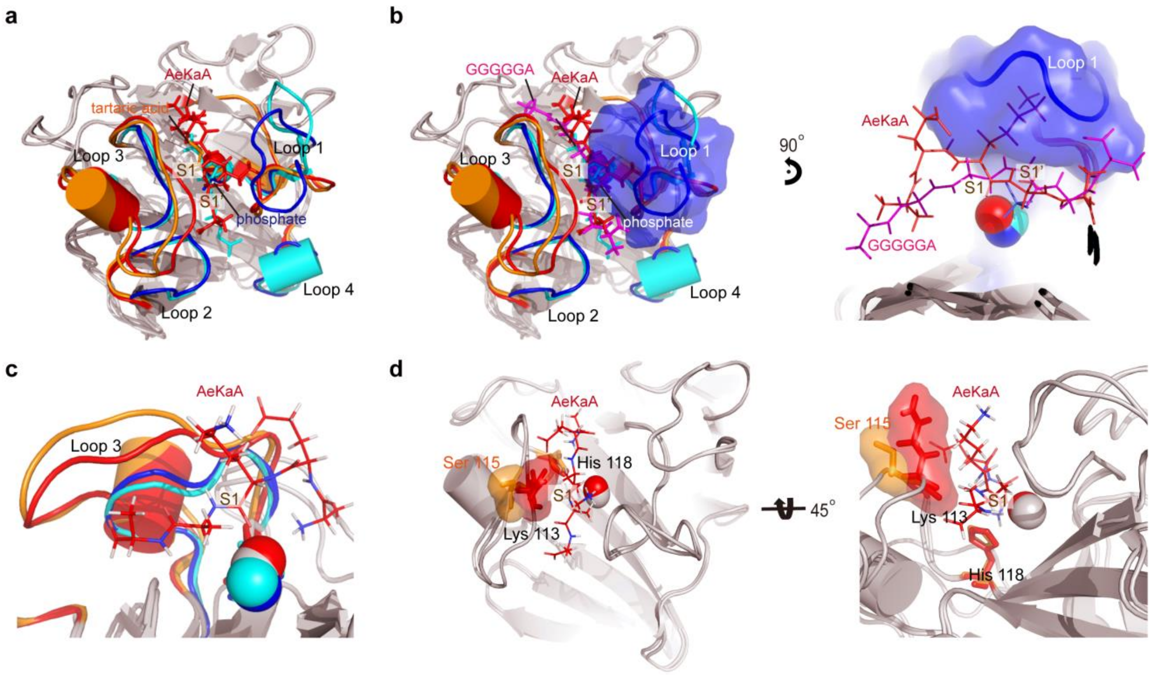

Supplementary Figure 9 | Comparison of the catalytic cavities of pseudoalterin, LasA, LytM, and lysostaphin. a, Structural superposition of pseudoalterin with LasA, LytM, and lysostaphin.

The loops and ligands of pseudoalterin, LasA, LytM, and lysostaphin are colored in red (loop1: 17-32, loop2: 61-63, loop3: 101-117, loop4: 127-129), orange (loop1: 19-34, loop2: 63-65, loo3: 103-119, loop4: 129-131), cyan (loop1:202-213, loop2: 237-242, loop3: 280-290, loo4: 298-305) and blue (loop1: 269-282, loop2: 305-311, loo3: 349-358, loop4: 367-374), respectively. b, The surface view of the loop 1 of lysostaphin. The big loop 1 makes the catalytic cavity of lysostaphin less wide-open than that of pseudoalterin. c and d, Comparison of the S1 pockets of pseudoalterin, LasA, LytM, and lysostaphin. Pseudoalterin, LasA, LytM, and lysostaphin are colored in red, orange, cyan and blue, respectively. The depth of the S1 pocket of pseudoalterin is mainly defined by the side chain of His118 and the main chain of loop 3 (b) and can be influenced by the side chain of loop 3, especially the side chain of Q113 (c). Pseudoalterin has the deepest S1 pocket among these enzymes.

a

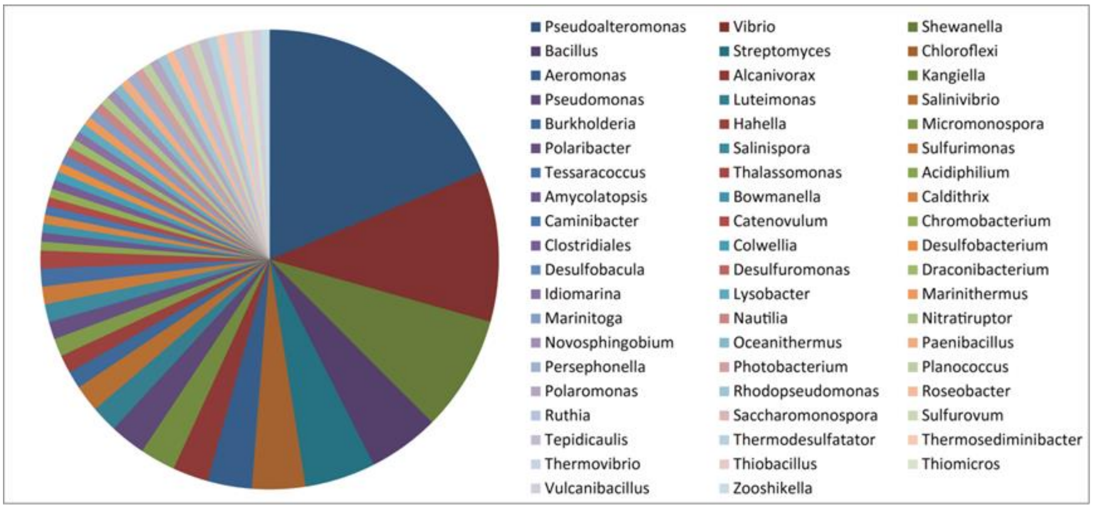

b

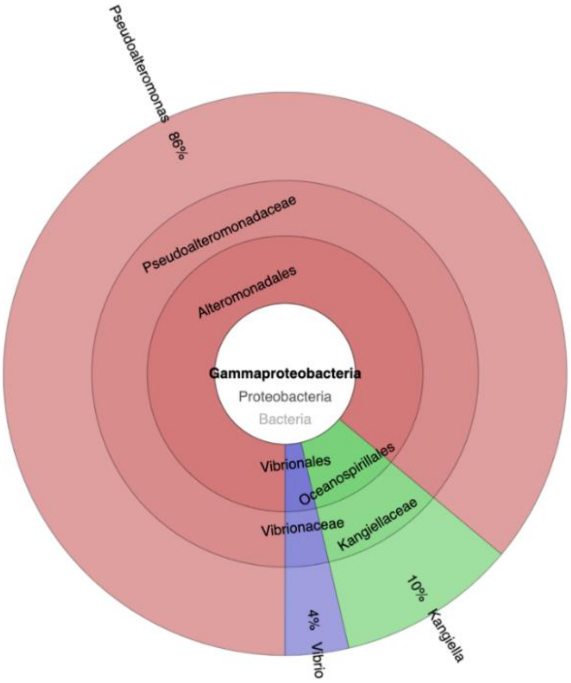

Supplementary Figure 10 | Analysis of the distribution of pseudoalterin-like protease. a, Statistical analysis of bacteria containing pseudoalterin-like protease from seawater, marine sediments, hydrothermal vents, sea ice and marine animals. b, Phylogenetic distribution of pseudoalterin-like protease sequences retrieved from the Tara Oceans Microbiome databases.

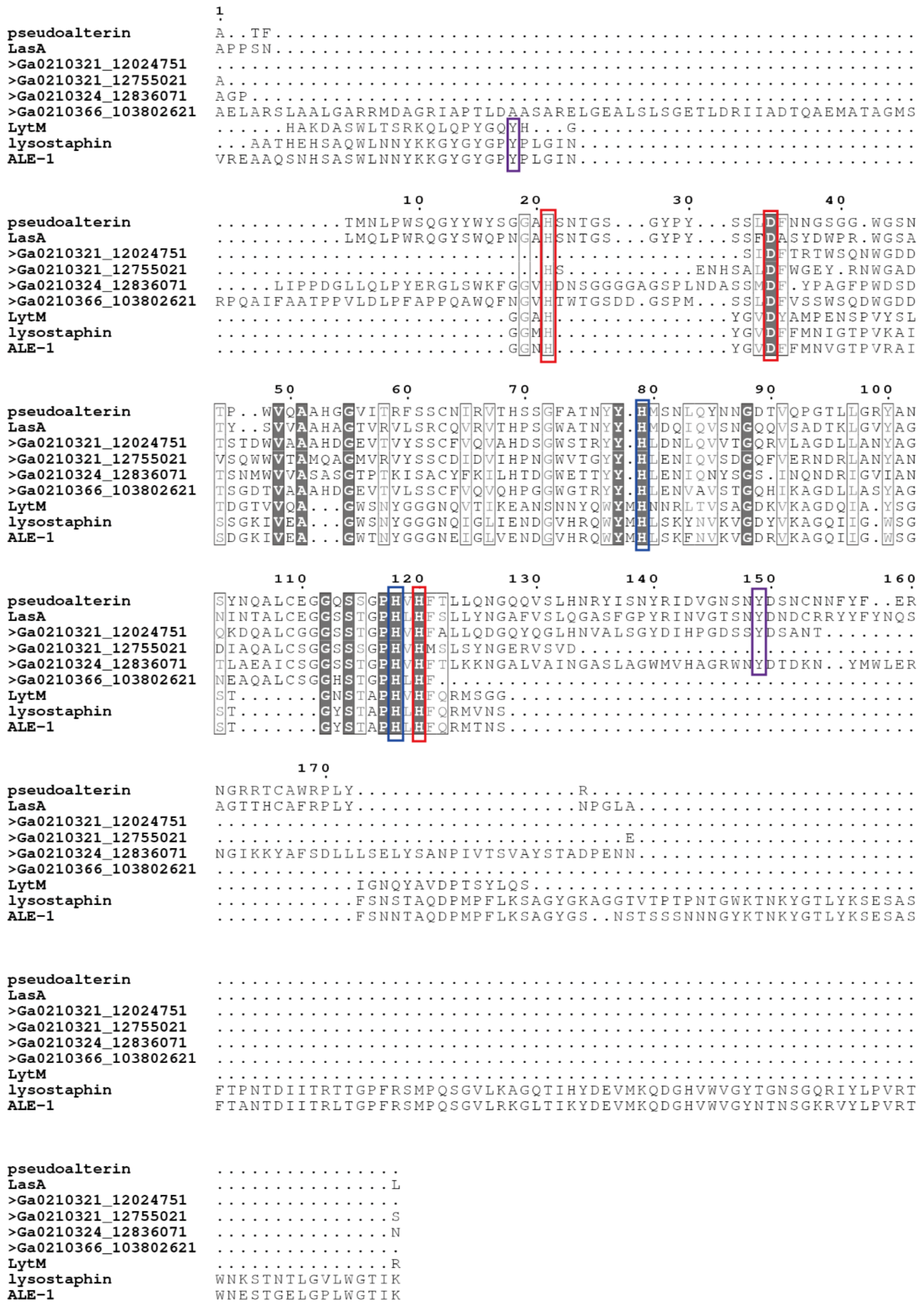

Supplementary Figure 11 | Multiple sequence alignment of pseudoalterin-like protease retrieved from metatranscriptome. Zinc metal coordination residues are highlighted in red boxes; Catalytic residues are highlighted in blue boxes. Residues that interact with zinc ion through water molecules are highlighted in purple boxes. The water molecules occupied the substrates binding positions.

Supplementary Table 1 | Killing activity of pseudoalterin against various marine bacteria with different PG chemotypes.

Strains	Peptide stem[a]	Peptide bridge[a]	killing rate (%)[b]
Staphylococcus warneri MCCC 1A00423 (+)[c]	Ae(q)Ka[1]	GGGGG[1]	84.02±0.92
Exiguobacterium profundum MCCC 1A04006 (+)	Ae(q)Ka[2]	G[2]	79.01±0.2
Exiguobacterium sp. MCCC 1A08510 (+)	Ae(q)Ka[2]	G[2]	83.34±0.65
Exiguobacterium profundum MCCC 1A09411 (+)	Ae(q)Ka[2]	G[2]	72.61±4.49
Exiguobacterium sp. MCCC 1A00445 (+)	Ae(q)Ka[2]	G[2]	90.72±0.17
Micrococcus luteus MCCC 1A04032 (+)	Ae(q)Ka/Ae(G)Ka[3]	EG/Ae(G)Ka[3]	44.32±1.18
Bacillus infantis MCCC 1A06626 (+)	Ae(q)Ka/AeKa/AeKa[3]	G/d(n)/Ad[3]	71.81±0.98
Bacillus sp. CF12-9 (+)	Ae(q)Ka/AeKa/AeKa[3]	G/d(n)/Ad[3]	16.94±0.63
Sporosarcina pasteurii MCCC 1A08736 (+)	AeKa[3,4]	Ge[3,4]	91.32±0.16
Sporosarcina aquimarina MCCC 1A05863 (+)	AeKa[3,4]	Ge[3,4]	25.57±1.92
Streptomyces sp. MCCC 1A06664 (+)	Ae(q)pa[3,5]	G[3,5]	29.2±1.92
Sanguibacter inulinus MCCC 1A05807 (+)	Ae(q)Ka[3,6]	Se[3,6]	50.18±1.6
Ornithinimicrobium kibberense MCCC 1A05447 (+)	Ae(q)Oa[3,7]	dGA[3,6]	88.65±0.07
Halobacillus mangrovi MCCC 1A04641 (+)	Ae(q)Oa[8]	d[7]	23.53±1.12
Pontibacillus sp. MCCC 1A04056 (+)	Ae(q)Pa[9]	-	60.14±0.19
Mycobacterium poriferae MCCC 1A09300 (+)	Ae(q)Pa[3]	-	-
Mycobacterium poriferae MCCC 1A05895 (+)	Ae(q)Pa[3]	-	-
Mycobacterium psychrotolerans MCCC 1A01315 (+)	Ae(q)Pa[3]	-	-
Microbacterium esteraromaticum MCCC 1A04803 (+)	Ge(Hyg)K(L-Hsr)a[10]	GK/Go[9]	-
Arthrobacter mysorens MCCC 1A05493 (+)	SeOa/Ge(hyg)Ea/Ae(a/q)Ka[3]	k/o/GGK/TA[3]	-
Marinibacillus sp. MCCC 1A04019 (+)	Ae(q)Ka[11]	-	-
Haliea mediterranea 1K01502 (-)[d]	Ae(q)Pa[1,3]	-	-
Hyphomonas beringensis 1K01528 (-)	Ae(q)Pa[1,3]	-	-
Pantoea agglomerans 1K01697 (-)	Ae(q)Pa[1,3]	-	-
Pseudomonas stutzeri 1K02121 (-)	Ae(q)Pa[1,3]	-	-

[a] The peptide stem and bridge of each genus are cited from the indicated references.

[b] The suspension of bacterial cells (OD_{600} = 0.8~1.0) was incubated at 25°C for 120 min with 20 μg ml^{-1} of pseudoalterin in 20 mM Tris-HCI buffer (pH 9.0). The killing rate (%) was calculated as the percentage of the decrease of OD_{600}/the initial OD_{600}. Data are from triplicate experiments (mean ± S.D.). Source data are provided as a Source Data file.

[c](+), Gram-positive bacteria.

[d](-), Gram-negative bacteria.

Supplementary Table 2 | Plasmids constructed in this study.

Plasmid	Phenotype and genotype
pMT*psn*	knockout vector for pseudoalterin, pMT carrying the upstream and downstream homologous arms of *psn*
pK18*gspE*	knockout vector for GspE, pK18*mobsacB*-Ery carrying the upstream and downstream homologous arms of *gspE*
pEV*psn*	complementary plasmid for Δ*psn*, pEV carrying the coding sequence of pseudoalterin
pEV*gspE*	complementary plasmid for Δ*gspE*, pEV carrying the coding sequence of GspE

Supplementary Table 3 | Strains constructed in this study.

Strain	Phenotype
Δ*psn*	*pseudoalterin* gene deletion mutant of CF6-2
Δ*gspE*	*gspE* gene deletion mutant of CF6-2
Δ*psn/*pEV*psn*	the complementary strain of Δ*psn*
Δ*gspE/*pEV*gspE*	the complementary strain of Δ*gspE*

Supplementary References

1 Vollmer, W., Blanot, D. & De Pedro, M. Peptidoglycan structure and architecture. *Fems Microbiol. Rev.* **32**, 149-167 (2008).

2 Mohan Kulshreshtha, N., Kumar, R., Begum, Z., Shivaji, S. & Kumar, A. *Exiguobacterium alkaliphilum* sp. nov. isolated from alkaline wastewater drained sludge of a beverage factory. *Int J Syst Evol Microbiol*. **63**, 4374-4379 (2013).

3 Schleifer, K. H. & Kandler, O. Peptidoglycan types of bacterial cell walls and their taxonomic implications. *Bacteriol. Rev.* **36**, 407-477 (1972).

4 Wolfgang, W. J. et al. *Sporosarcina newyorkensis* sp. nov. from clinical specimens and raw cow's milk. *Int J Syst Evol Microbiol*. **62**, 322-329 (2012).

5 Nguyen, T. M. & Kim, J. Antifungal and antibacterial activities of *streptomyces polymachus* sp. nov. isolated from soil. *Int J Syst Evol Microbiol*. **65**, 2385-2390 (2015).

6. Pascual, C., Collins, M. D., Grimont, P. A., Dominguez, L., & Fernandez-garayzabal, J. F. *Sanguibacter inulinus* sp. nov. *Int J Syst Evol Microbiol.* **46**(3), 811. (1996).

7 Groth, I., Schumann, P., Weiss, N., Schuetze, B. & Stackebrandt, E. *Ornithinimicrobium humiphilum* gen. nov. sp. nov. a novel soil actinomycete with L-ornithine in the peptidoglycan. *Int J Syst Evol Microbiol*. **51**, 81-87 (2001).

8 Yoon, J. H., Kang, S. J., Jung, Y. T., & Oh, T. K. *Halobacillus campisalis* sp. nov. containing meso-diaminopimelic acid in the cell-wall peptidoglycan, and emended description of the genus *Halobacillus*. *Int J Syst Evol Microbiol*. **57**, 2021-2025 (2007).

9 Lim, J. M. et al. *Pontibacillus marinus* sp. nov. a moderately halophilic bacterium from a solar saltern, and emended description of the genus *Pontibacillus*. *Int J Syst Evol Microbiol*. **55**, 1027-1031 (2005).

10 Brennan, P. J. Structure, function, and biogenesis of the cell wall of *Mycobacterium tuberculosis*. *Tuberculosis (Edinb).* **83**, 91-97 (2003).

11 Yoon, J. H., Kim, I. G., Schumann, P., Oh, T. K., & Park, Y. H. *Marinibacillus campisalis* sp nov. a moderate halophile isolated from a marine solar saltern in korea, with emended description of the genus *Marinibacillus*. *Int J Syst Evol Microbiol*. **54**, 1317-1321 (2004).
