# Supplementary material for: A predator-prey interaction between a marine Pseudoalteromonas sp. and Gram-positive bacteria
Source: Nat Commun. 2020 Jan 15;11:285. doi: 10.1038/s41467-019-14133-x (PMC6962226; doi:10.1038/s41467-019-14133-x)
Supplement: Supplementary file 2 — Description of Additional Supplementary Files [file 41467_2019_14133_MOESM2_ESM.pdf]

Description of Additional Supplementary Files

File Name: Supplementary Movie 1
Description: The observation was monitored under atomic force microscopy in liquid conditions. The image size was 10 μm × 3.3 μm.

File Name: Supplementary Data 1
Description: Bacteria containing pseudoalterin-like protease from seawater, marine sediment and hydrothermal vent.

File Name: Supplementary Data 2
Description: Primers used in this study.

File Name: Supplementary Data 3
Description: Coastal and marine sediment metatranscriptomics datasets obtained from the Integrated Microbial Genomes/Microbiomes database.
