# Supplementary figures and images for: A predator-prey interaction between a marine Pseudoalteromonas sp. and Gram-positive bacteria
Source: Nat Commun. 2020 Jan 15;11:285. doi: 10.1038/s41467-019-14133-x (PMC6962226; doi:10.1038/s41467-019-14133-x)

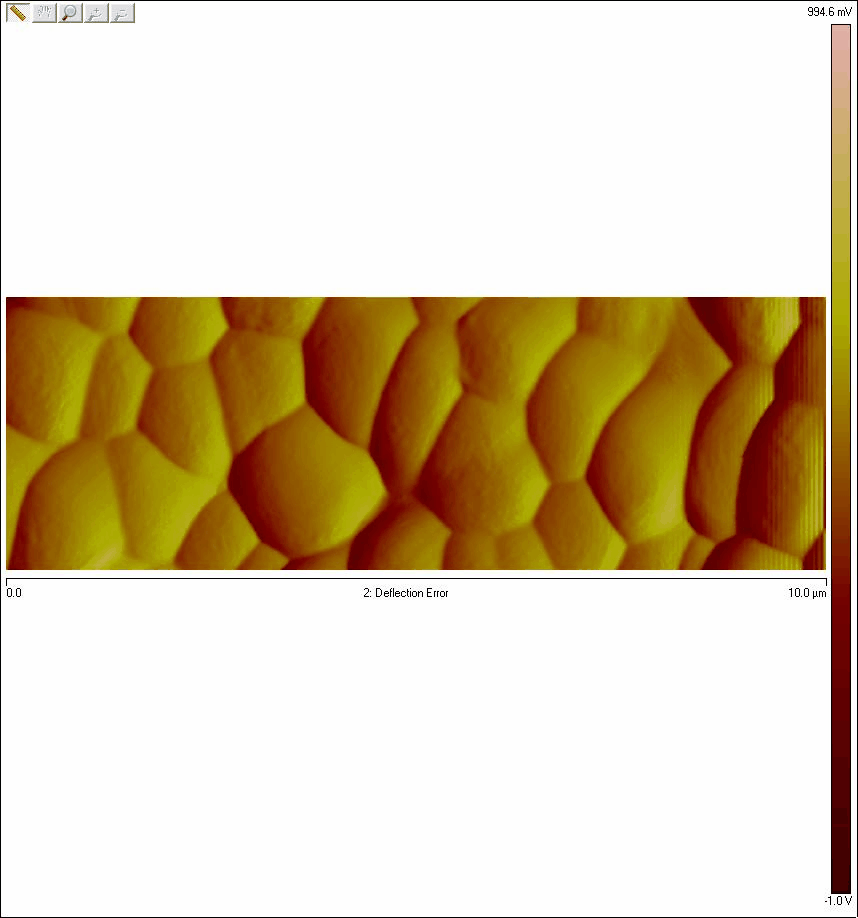

Supplement: Supplementary file 3 — Supplementary Movie 1 [file 41467_2019_14133_MOESM3_ESM.gif]
