# Supplementary material for: A predator-prey interaction between a marine Pseudoalteromonas sp. and Gram-positive bacteria
Source: Nat Commun. 2020 Jan 15;11:285. doi: 10.1038/s41467-019-14133-x (PMC6962226; doi:10.1038/s41467-019-14133-x)
Supplement: Supplementary file 7 — Reporting Summary [file 41467_2019_14133_MOESM7_ESM.pdf]

nature research

Corresponding author(s): *Double-blind peer review submissions: write DBPR and your manuscript number here instead of author names.*

Last updated by author(s): *YYYY-MM-DD*

Reporting Summary

Nature Research wishes to improve the reproducibility of the work that we publish. This form provides structure for consistency and transparency in reporting. For further information on Nature Research policies, see Authors & Referees and the Editorial Policy Checklist .

Statistics

For all statistical analyses, confirm that the following items are present in the figure legend, table legend, main text, or Methods section.

n/a	Confirmed	
x		The exact sample size (*n*) for each experimental group/condition, given as a discrete number and unit of measurement
	x	A statement on whether measurements were taken from distinct samples or whether the same sample was measured repeatedly
	x	The statistical test(s) used AND whether they are one- or two-sided<br>*Only common tests should be described solely by name; describe more complex techniques in the Methods section.*
x		A description of all covariates tested
x		A description of any assumptions or corrections, such as tests of normality and adjustment for multiple comparisons
x		A full description of the statistical parameters including central tendency (e.g. means) or other basic estimates (e.g. regression coefficient) AND variation (e.g. standard deviation) or associated estimates of uncertainty (e.g. confidence intervals)
x		For null hypothesis testing, the test statistic (e.g. *F*, *t*, *r*) with confidence intervals, effect sizes, degrees of freedom and *P* value noted<br>*Give P values as exact values whenever suitable.*
x		For Bayesian analysis, information on the choice of priors and Markov chain Monte Carlo settings
x		For hierarchical and complex designs, identification of the appropriate level for tests and full reporting of outcomes
x		Estimates of effect sizes (e.g. Cohen's *d*, Pearson's *r*), indicating how they were calculated

Our web collection on statistics for biologists contains articles on many of the points above.

Software and code

Policy information about availability of computer code

Data collection

The x-ray diffraction data of the crystals were collected using HKL2000.
AFM imaging data were collected using Nanoscope v. 8.10.
SEM imaging data were collected using xT microscope Control v6.2.4 build 3069.
The circular dichroism spectra were monitoried using JASCO Spectra Manager.
The western blots were collected using MYImage Analysis Software.
The data of absorbance were collected using Soft Max Pro.
The data of qPCR were collected using the LightCycler® 480 software.

Data analysis

The crystals structures were analyzed using PyMol.
The data of qPCR were analyzed using the LightCycler® 480 software.
The data of HPLC were analyzed using Chromeleon.
The data of LC-MS were analyzed using Data Analysis software.
The sequences of the released peptides were analyzed by using ExPASy tools.
The data were analyzed using Origin.

For manuscripts utilizing custom algorithms or software that are central to the research but not yet described in published literature, software must be made available to editors/reviewers. We strongly encourage code deposition in a community repository (e.g. GitHub). See the Nature Research guidelines for submitting code & software for further information.

Data

Policy information about availability of data

All manuscripts must include a data availability statement. This statement should provide the following information, where applicable:

- Accession codes, unique identifiers, or web links for publicly available datasets
- A list of figures that have associated raw data
- A description of any restrictions on data availability

Accession codes of the structure: 6IK4.
The data that support the findings of this study are available from the corresponding author on reasonable request.

Field-specific reporting

Please select the one below that is the best fit for your research. If you are not sure, read the appropriate sections before making your selection.

☒ Life sciences ☐ Behavioural & social sciences ☐ Ecological, evolutionary & environmental sciences

For a reference copy of the document with all sections, see nature.com/documents/nr-reporting-summary-flat.pdf

Life sciences study design

All studies must disclose on these points even when the disclosure is negative.

Sample size	N/A
Data exclusions	N/A
Replication	All experiments were repeated 3 times as completely independent experiments unless otherwise is stated.
Randomization	N/A
Blinding	N/A

Reporting for specific materials, systems and methods

We require information from authors about some types of materials, experimental systems and methods used in many studies. Here, indicate whether each material, system or method listed is relevant to your study. If you are not sure if a list item applies to your research, read the appropriate section before selecting a response.

Materials & experimental systems

n/a	Involved in the study	
☐	☒	Antibodies
☒	☐	Eukaryotic cell lines
☒	☐	Palaeontology
☒	☐	Animals and other organisms
☒	☐	Human research participants
☒	☐	Clinical data

Methods

n/a	Involved in the study	
☒	☐	ChIP-seq
☒	☐	Flow cytometry
☒	☐	MRI-based neuroimaging

Antibodies

Antibodies used	A pseudoalterin polyclonal antibody (1000× dillusion of 3.4 mg ml-1) prepared by the Beijing Genomics Institute, China.<br>A horseradish peroxidase-conjugated secondary antibody (1000× dillusion of 2 mg ml-1) produced by Abcam, UK.
Validation	The pseudoalterin antibody concentration is 3.4 mg ml-1 and the purity of the antibody is above 90%.<br>The horseradish peroxidase-conjugated secondary antibody concentration is 2 mg ml-1 and its catalogue number is goat anti-rabbit ab6721.
